# Supplementary material for: tRNA Core Hypothesis for the Transition from the RNA World to the Ribonucleoprotein World
Source: Life (Basel). 2016 Mar 23;6(2):15. doi: 10.3390/life6020015 (PMC4931452; doi:10.3390/life6020015)
Supplement: Supplementary file 1 [file life-06-00015-s001.zip › Supplementary information 2.pdf]

Supplementary Materials: tRNA Core Hypothesis for the Transition from the RNA World to the Ribonucleoprotein World

Savio T. de Farias [1,†,*], Thais G. Rêgo [2,†] and Marco V. José [3,†,*]

```
BLASTX 2.2.27+
Reference: Stephen F. Altschul, Thomas L. Madden, Alejandro
A. Schaffer, Jinghui Zhang, Zheng Zhang, Webb Miller, and
David J. Lipman (1997), "Gapped BLAST and PSI-BLAST: a new
generation of protein database search programs", Nucleic
Acids Res. 25:3389-3402.


RID: G67NCMY9013


Database: Non-redundant UniProtKB/SwissProt sequences
 454,086 sequences; 169,309,753 total letters



Query= 1

Length=121


 Score     E
Sequences producing significant alignments:                       (Bits)  Value

sp|A8ABM5.1|EF1A_IGNH4  RecName: Full=Elongation factor 1-alph...  29.3    1.2

ALIGNMENTS
>sp|A8ABM5.1|EF1A_IGNH4 RecName: Full=Elongation factor 1-alpha; Short=EF-1-
alpha; AltName:
Full=Elongation factor Tu; Short=EF-Tu
Length=442

 Score = 29.3 bits (64),  Expect = 1.2, Method: Composition-based stats.
 Identities = 15/38 (39%), Positives = 24/38 (63%), Gaps = 5/38 (13%)
 Frame = -1

Query  103  SSLAVGFEP-----THSVNSRIFQLQQGVEPVLARVPE  5
 S++AVG+ P      T SV SRI +++Q ++P   +V E
Sbjct  341  SAIAVGYTPVIHAHTASVASRIIEIKQKIDPRTGKVIE  378




Query= 2

Length=136


 Score     E
Sequences producing significant alignments:                       (Bits)  Value

sp|A8ABM5.1|EF1A_IGNH4  RecName: Full=Elongation factor 1-alph...  28.1    3.8

sp|Q29293.1|RL3_PIG  RecName: Full=60S ribosomal protein L3        26.6    8.4
```

```
ALIGNMENTS
>sp|A8ABM5.1|EF1A_IGNH4 RecName: Full=Elongation factor 1-alpha; Short=EF-1-
alpha; AltName:
Full=Elongation factor Tu; Short=EF-Tu
Length=442

 Score = 28.1 bits (61),  Expect = 3.8, Method: Composition-based stats.
 Identities = 17/44 (39%), Positives = 25/44 (57%), Gaps = 7/44 (16%)
 Frame = -1

Query  121  IWYGPAHHFAVGFEP-----THSVNSRIFQLQQGVEPVLARVPE  5
            IW+  A   AVG+ P      T SV SRI +++Q ++P   +V E
Sbjct  337  IWHPSA--IAVGYTPVIHAHTASVASRIIEIKQKIDPRTGKVIE  378


>sp|Q29293.1|RL3_PIG RecName: Full=60S ribosomal protein L3
Length=130

 Score = 26.6 bits (57),  Expect = 8.4, Method: Compositional matrix adjust.
 Identities = 10/19 (53%), Positives = 13/19 (68%), Gaps = 0/19 (0%)
 Frame = +2

Query  47   LEDSAVNRMGRFESYGEVV  103
            L D ++N +GRF  YGEV
Sbjct  106  LSDKSINPLGRFVHYGEVT  124



Query= 3

Length=138


                                                                      Score     E
Sequences producing significant alignments:                          (Bits)  Value


sp|B3PIT8.1|MNMG_CELJU  RecName: Full=tRNA uridine 5-carboxyme...  27.3    6.7


>sp|B3PIT8.1|MNMG_CELJU RecName: Full=tRNA uridine 5-carboxymethylaminomethyl
modification
enzyme MnmG; AltName: Full=Glucose-inhibited division
protein A
Length=638

 Score = 27.3 bits (59),  Expect = 6.7, Method: Composition-based stats.
 Identities = 12/20 (60%), Positives = 15/20 (75%), Gaps = 0/20 (0%)
 Frame = +1

Query  70   SLKGEPSSGEYACPQVRVES  129
            SLKGEP +GE A  QV +E+
Sbjct  525  SLKGEPLAGEAAAEQVEIEA  544
```

```
Query= 4

Length=148


                                                                      Score     E
Sequences producing significant alignments:                          (Bits)  Value

sp|B1I165.1|KTHY_DESAP  RecName: Full=Thymidylate kinase; AltN...  27.3    4.8


ALIGNMENTS
>sp|B1I165.1|KTHY_DESAP RecName: Full=Thymidylate kinase; AltName: Full=dTMP
kinase
Length=221

 Score = 27.3 bits (59),  Expect = 4.8, Method: Compositional matrix adjust.
 Identities = 12/27 (44%), Positives = 18/27 (67%), Gaps = 0/27 (0%)
 Frame = -3

Query  116  NSRIFQLQRSGGTQPGPADRRILLNSG  36
            N R+   +  GGT+ G A RR+LL++G
Sbjct  32   NHRVLVTREPGGTRIGEAVRRVLLDTG  58




Query= 5

Length=138


                                                                      Score     E
Sequences producing significant alignments:                          (Bits)  Value


sp|Q8Y003.1|RGMG_RALSO  RecName: Full=Putative ribose/galactos...  27.7    5.0

ALIGNMENTS

>sp|Q8Y003.1|RGMG_RALSO RecName: Full=Putative ribose/galactose/methyl
galactoside import

ATP-binding protein
Length=518

 Score = 27.7 bits (60),  Expect = 5.0, Method: Compositional matrix adjust.
 Identities = 11/28 (39%), Positives = 16/28 (57%), Gaps = 0/28 (0%)
 Frame = -1

Query  120  PWQVMSELIWVRRDSTRTCGQAYSPELR  37
 P+  ++E IW+RR+    CG     ELR
Sbjct  116  PYMTVAENIWIRREPRNRCGLVDHAELR  143
```

```
Query= 7

Length=131


                                                                      Score     E
Sequences producing significant alignments:                          (Bits)  Value

sp|Q2NQF4.1|DAPF_SODGM  RecName: Full=Diaminopimelate epimeras...  26.6    8.4

ALIGNMENTS
>sp|Q2NQF4.1|DAPF_SODGM RecName: Full=Diaminopimelate epimerase; Short=DAP
epimerase
Length=274

 Score = 26.6 bits (57),  Expect = 8.4, Method: Compositional matrix adjust.
 Identities = 13/31 (42%), Positives = 16/31 (52%), Gaps = 0/31 (0%)
 Frame = -3

Query  102  SPPLVRRDSTRTCGQAYSPELRGSNPY*PEF  10
            SP ++RR S R CG  +   L    PY PE
Sbjct  26   SPEMIRRLSDRHCGVGFDQLLVVEPPYDPEL  56




Query= 8

Length=133


                                                                      Score     E
Sequences producing significant alignments:                          (Bits)  Value


sp|Q8EDG1.1|CDD_SHEON  RecName: Full=Cytidine deaminase; AltNa...  28.1    2.9
sp|Q0HHI5.1|CDD_SHESM  RecName: Full=Cytidine deaminase; AltNa...  28.1    2.9
sp|Q0HTT9.1|CDD_SHESR  RecName: Full=Cytidine deaminase; AltNa...  28.1    2.9
sp|A0KYD1.1|CDD_SHESA  RecName: Full=Cytidine deaminase; AltNa...  28.1    2.9
sp|A8H5H1.1|CDD_SHEPA  RecName: Full=Cytidine deaminase; AltNa...  27.7    4.4

ALIGNMENTS

>sp|Q8EDG1.1|CDD_SHEON RecName: Full=Cytidine deaminase; AltName: Full=Cytidine
aminohydrolase;
Short=CDA
Length=296

 Score = 28.1 bits (61),  Expect = 2.9, Method: Composition-based stats.
 Identities = 14/35 (40%), Positives = 21/35 (60%), Gaps = 0/35 (0%)
 Frame = -3

Query  125  LQGPSAVRDVILNAERIERPWQVMSELI*GGRTRI  21
            L G S + D+I+NA       Q M+EL+ GG+ +I
Sbjct  112  LSGESQIVDMIVNASPCGHCRQFMNELVDGGQIKI  146


>sp|Q0HHI5.1|CDD_SHESM RecName: Full=Cytidine deaminase; AltName: Full=Cytidine
aminohydrolase;
Short=CDA
Length=296

 Score = 28.1 bits (61),  Expect = 2.9, Method: Composition-based stats.
 Identities = 14/35 (40%), Positives = 21/35 (60%), Gaps = 0/35 (0%)
```

```
 Frame = -3

Query  125  LQGPSAVRDVILNAERIERPWQVMSELI*GGRTRI  21
            L G S + D+I+NA       Q M+EL+ GG+ +I
Sbjct  112  LSGESQIVDMIVNASPCGHCRQFMNELVEGGQIKI  146


>sp|Q0HTT9.1|CDD_SHESR RecName: Full=Cytidine deaminase; AltName: Full=Cytidine
aminohydrolase;
Short=CDA
Length=296

 Score = 28.1 bits (61),  Expect = 2.9, Method: Composition-based stats.
 Identities = 14/35 (40%), Positives = 21/35 (60%), Gaps = 0/35 (0%)
 Frame = -3

Query  125  LQGPSAVRDVILNAERIERPWQVMSELI*GGRTRI  21
            L G S + D+I+NA       Q M+EL+ GG+ +I
Sbjct  112  LSGESQIVDMIVNASPCGHCRQFMNELVEGGQIKI  146


>sp|A0KYD1.1|CDD_SHESA RecName: Full=Cytidine deaminase; AltName: Full=Cytidine
aminohydrolase;
Short=CDA
Length=296

 Score = 28.1 bits (61),  Expect = 2.9, Method: Composition-based stats.
 Identities = 14/35 (40%), Positives = 21/35 (60%), Gaps = 0/35 (0%)
 Frame = -3

Query  125  LQGPSAVRDVILNAERIERPWQVMSELI*GGRTRI  21
            L G S + D+I+NA       Q M+EL+ GG+ +I
Sbjct  112  LSGESQIVDMIVNASPCGHCRQFMNELVEGGQIKI  146


>sp|A8H5H1.1|CDD_SHEPA RecName: Full=Cytidine deaminase; AltName: Full=Cytidine
aminohydrolase;
Short=CDA
Length=296

 Score = 27.7 bits (60),  Expect = 4.4, Method: Composition-based stats.
 Identities = 13/35 (37%), Positives = 20/35 (57%), Gaps = 0/35 (0%)
 Frame = -3

Query  125  LQGPSAVRDVILNAERIERPWQVMSELI*GGRTRI  21
            L G S + D+I+NA       Q ++EL+ GG+  I
Sbjct  112  LSGESIIEDIIVNASPCGHCRQFINELVDGGKVNI  146
```

```
Query= 9

Length=125


                                                                      Score     E
Sequences producing significant alignments:                          (Bits)  Value

sp|P06221.1|RPOL_BPSP6  RecName: Full=DNA-directed RNA polymerase  28.1    3.7

ALIGNMENTS
>sp|P06221.1|RPOL_BPSP6 RecName: Full=DNA-directed RNA polymerase
Length=874

 Score = 28.1 bits (61),  Expect = 3.7, Method: Composition-based stats.
 Identities = 14/27 (52%), Positives = 16/27 (59%), Gaps = 0/27 (0%)
 Frame = -2

Query  115  SAVQVSSHELQNESNDLGKLCLNLSEG  35
            S V V S  L  +SNDLGK  L  +EG
Sbjct  414  SRVYVQSSTLSPQSNDLGKALLRFTEG  440




Query= 10

Length=111


                                                                      Score     E
Sequences producing significant alignments:                          (Bits)  Value

sp|Q8FS33.1|RPOA_COREF  RecName: Full=DNA-directed RNA polymer...  26.9    6.2
sp|A4QBQ7.1|RPOA_CORGB  RecName: Full=DNA-directed RNA polymer...  26.6    8.0
sp|Q8NSV2.1|RPOA_CORGL  RecName: Full=DNA-directed RNA polymer...  26.6    8.2
sp|Q6NJ63.1|RPOA_CORDI  RecName: Full=DNA-directed RNA polymer...  26.6    9.0

ALIGNMENTS
>sp|Q8FS33.1|RPOA_COREF RecName: Full=DNA-directed RNA polymerase subunit alpha;
Short=RNAP
subunit alpha; AltName: Full=RNA polymerase subunit alpha;
AltName: Full=Transcriptase subunit alpha
Length=338

 Score = 26.9 bits (58),  Expect = 6.2, Method: Composition-based stats.
 Identities = 12/22 (55%), Positives = 15/22 (68%), Gaps = 0/22 (0%)
 Frame = -3

Query  85  VRDVILNCRGFVQSSQGVEPVL  20
           V D+ILN +G V SS   EPV+
Sbjct  73  VSDIILNIKGLVLSSDSDEPVI  94


>sp|A4QBQ7.1|RPOA_CORGB RecName: Full=DNA-directed RNA polymerase subunit alpha;
Short=RNAP
subunit alpha; AltName: Full=RNA polymerase subunit alpha;
AltName: Full=Transcriptase subunit alpha
Length=338

 Score = 26.6 bits (57),  Expect = 8.0, Method: Composition-based stats.
 Identities = 12/25 (48%), Positives = 17/25 (68%), Gaps = 0/25 (0%)
 Frame = -3
```

```
Query  85  VRDVILNCRGFVQSSQGVEPVLARV  11
           V D+ILN +G V SS   EPV+ ++
Sbjct  73  VSDIILNIKGLVLSSDSDEPVVMQL  97


>sp|Q8NSV2.1|RPOA_CORGL RecName: Full=DNA-directed RNA polymerase subunit alpha; 
Short=RNAP
subunit alpha; AltName: Full=RNA polymerase subunit alpha;
AltName: Full=Transcriptase subunit alpha
Length=338

 Score = 26.6 bits (57),  Expect = 8.2, Method: Composition-based stats.
 Identities = 12/25 (48%), Positives = 17/25 (68%), Gaps = 0/25 (0%)
 Frame = -3

Query  85  VRDVILNCRGFVQSSQGVEPVLARV  11
           V D+ILN +G V SS   EPV+ ++
Sbjct  73  VSDIILNIKGLVLSSDSDEPVVMQL  97


>sp|Q6NJ63.1|RPOA_CORDI RecName: Full=DNA-directed RNA polymerase subunit alpha; 
Short=RNAP
subunit alpha; AltName: Full=RNA polymerase subunit alpha;
AltName: Full=Transcriptase subunit alpha
Length=338

 Score = 26.6 bits (57),  Expect = 9.0, Method: Composition-based stats.
 Identities = 12/22 (55%), Positives = 15/22 (68%), Gaps = 0/22 (0%)
 Frame = -3

Query  85  VRDVILNCRGFVQSSQGVEPVL  20
           V D+ILN +G V SS   EPV+
Sbjct  73  VSDIILNIKGLVLSSDSDEPVV  94



Query= 12
Length=126


                                                                   Score     E
Sequences producing significant alignments:                       (Bits)  Value

sp|P49327.3|FAS_HUMAN  RecName: Full=Fatty acid synthase; Incl...  28.5    2.6

ALIGNMENTS
>sp|P49327.3|FAS_HUMAN RecName: Full=Fatty acid synthase; Includes: RecName: 
Full=[Acyl-carrier-protein]
S-acetyltransferase; Includes: RecName:
Full=[Acyl-carrier-protein] S-malonyltransferase; Includes:
RecName: Full=3-oxoacyl-[acyl-carrier-protein] synthase; Includes:
RecName: Full=3-oxoacyl-[acyl-carrier-protein] reductase;
Includes: RecName: Full=3-hydroxyacyl-[acyl-carrier-protein]
dehydratase; Includes: RecName: Full=Enoyl-[acyl-carrier-protein]
reductase; Includes: RecName: Full=Oleoyl-[acyl-carrier-protein]
hydrolase
Length=2511

 Score = 28.5 bits (62),  Expect = 2.6, Method: Compositional matrix adjust.
 Identities = 12/27 (44%), Positives = 17/27 (63%), Gaps = 0/27 (0%)
 Frame = -3

Query  124   FTLQGPSAVRDVILNRWLRSIWYGPAH  44
             +TLQ  + V DV+++RWLR    G  H
```

```
Sbjct  1067  YTLQDKAQVADVVVSRWLRVTVAGGVH  1093


Query= 13

Length=131


                                                                      Score     E
Sequences producing significant alignments:                          (Bits)  Value

sp|Q03396.1|L_SV5WR  RecName: Full=RNA-directed RNA polymerase...  29.6    1.3

ALIGNMENTS
>sp|Q03396.1|L_SV5WR RecName: Full=RNA-directed RNA polymerase L; Short=Protein
L;
AltName: Full=Large structural protein; AltName: Full=Replicase;
AltName: Full=Transcriptase; Includes: RecName: Full=RNA-directed
RNA polymerase; Includes: RecName: Full=mRNA (guanine-N(7)-)-methyltransferase;
Includes: RecName: Full=mRNA
guanylyltransferase
Length=2255

 Score = 29.6 bits (65),  Expect = 1.3, Method: Composition-based stats.
 Identities = 15/30 (50%), Positives = 17/30 (57%), Gaps = 3/30 (10%)
 Frame = -3

Query  114  PGPADRRILLN---SDGFDPSGTAQPTTRG  34
            P P +RR+LLN    D FDPS   Q  T G
Sbjct  507  PNPFNRRLLLNFLGDDKFDPSVELQYVTSG  536



Query= 14

Length=125


                                                                      Score     E
Sequences producing significant alignments:                          (Bits)  Value

sp|Q9VQC4.1|GLCTK_DROME  RecName: Full=Glycerate kinase           28.5    2.2

ALIGNMENTS
>sp|Q9VQC4.1|GLCTK_DROME RecName: Full=Glycerate kinase
Length=487

 Score = 28.5 bits (62),  Expect = 2.2, Method: Compositional matrix adjust.
 Identities = 11/21 (52%), Positives = 15/21 (71%), Gaps = 0/21 (0%)
 Frame = -1

Query  86   LICSGHSAVQVSSHELGGRTR  24
            LIC G   ++VS H LGGR++
Sbjct  369  LICGGEPVIKVSGHGLGGRSQ  389




Query= 15

Length=148
```

```
                                                                      Score     E
Sequences producing significant alignments:                          (Bits)  Value


sp|Q4QLD1.1|HIS82_HAEI8  RecName: Full=Histidinol-phosphate am...  27.3    6.9

ALIGNMENTS


>sp|Q4QLD1.1|HIS82_HAEI8 RecName: Full=Histidinol-phosphate aminotransferase 2;
AltName:
Full=Imidazole acetol-phosphate transaminase 2
Length=366

 Score = 27.3 bits (59),  Expect = 6.9, Method: Composition-based stats.
 Identities = 14/39 (36%), Positives = 23/39 (59%), Gaps = 0/39 (0%)
 Frame = -1

Query  133  NPDLRTGVFS*TQGVEPVLARVPERLAVGFEPTHSVNSR  17
            NP+  TG F  +Q +E  LA+VPE + V  +  ++  +R
Sbjct  163  NPNNPTGNFLTSQEIEDFLAKVPENVIVVLDEAYTEFTR  201


Query= 16

Length=143


                                                                      Score     E
Sequences producing significant alignments:                          (Bits)  Value


sp|Q30ZW0.1|TPIS_DESDG  RecName: Full=Triosephosphate isomeras...  28.1    3.4

ALIGNMENTS

>sp|Q30ZW0.1|TPIS_DESDG RecName: Full=Triosephosphate isomerase; Short=TIM;
AltName:
Full=Triose-phosphate isomerase
Length=250

 Score = 28.1 bits (61),  Expect = 3.4, Method: Composition-based stats.
 Identities = 13/29 (45%), Positives = 20/29 (69%), Gaps = 1/29 (3%)
 Frame = -2

Query  103  LSEGVEPVLARV-PERLAVGFEPTHSVNS  20
            LSEG+  V A + PERLA+ +EP  ++ +
Sbjct  148  LSEGLSGVPAGIAPERLAIAYEPVWAIGT  176




Query= 17

Length=156


                                                                      Score     E
Sequences producing significant alignments:                          (Bits)  Value


sp|B3PIT8.1|MNMG_CELJU  RecName: Full=tRNA uridine 5-carboxyme...  27.3    7.5
```

```
ALIGNMENTS

>sp|B3PIT8.1|MNMG_CELJU RecName: Full=tRNA uridine 5-carboxymethylaminomethyl
modification
enzyme MnmG; AltName: Full=Glucose-inhibited division
protein A
Length=638

 Score = 27.3 bits (59),  Expect = 7.5, Method: Composition-based stats.
 Identities = 12/20 (60%), Positives = 15/20 (75%), Gaps = 0/20 (0%)
 Frame = +1

Query  88   SLKGEPSSGEYACPQVRVES  147
 SLKGEP +GE A  QV +E+
Sbjct  525  SLKGEPLAGEAAAEQVEIEA  544




Query= 18

Length=148


                                                                      Score     E
Sequences producing significant alignments:                          (Bits)  Value

sp|Q1AW66.1|PYRH_RUBXD  RecName: Full=Uridylate kinase; Short=...  27.3    5.7

ALIGNMENTS
>sp|Q1AW66.1|PYRH_RUBXD RecName: Full=Uridylate kinase; Short=UK; AltName:
Full=Uridine
monophosphate kinase; Short=UMP kinase; Short=UMPK
Length=255

 Score = 27.3 bits (59),  Expect = 5.7, Method: Composition-based stats.
 Identities = 15/35 (43%), Positives = 17/35 (49%), Gaps = 0/35 (0%)
 Frame = +3

Query  42   SNPTARVQENTPVRRSGLSPSGPPLRNSG*YGFDP  146
            S   A   E  P+RR  L  SG  L  +G YG DP
Sbjct  4    SARAAETSELGPLRRVVLKLSGESLAGNGGYGIDP  38


Query= 19

Length=134


                                                                      Score     E
Sequences producing significant alignments:                          (Bits)  Value



sp|Q29293.1|RL3_PIG  RecName: Full=60S ribosomal protein L3        26.9    4.5


ALIGNMENTS

>sp|Q29293.1|RL3_PIG RecName: Full=60S ribosomal protein L3
Length=130

 Score = 26.9 bits (58),  Expect = 4.5, Method: Compositional matrix adjust.
```

```
 Identities = 9/23 (39%), Positives = 15/23 (65%), Gaps = 0/23 (0%)
 Frame = +2

Query  8    LEDSAVNRMGRFESYGESSGEYA  76
            L D ++N +GRF  YGE + ++
Sbjct  106  LSDKSINPLGRFVHYGEVTNDFV  128




Query= 20

Length=129


                                                                      Score     E
Sequences producing significant alignments:                          (Bits)  Value

sp|Q5GS39.1|GPDA_WOLTR  RecName: Full=Glycerol-3-phosphate deh...  28.1    3.4
sp|P61749.1|GPDA_WOLPM  RecName: Full=Glycerol-3-phosphate deh...  26.9    6.5
sp|C0R3K2.1|GPDA_WOLWR  RecName: Full=Glycerol-3-phosphate deh...  26.9    6.6

ALIGNMENTS
>sp|Q5GS39.1|GPDA_WOLTR RecName: Full=Glycerol-3-phosphate dehydrogenase
[NAD(P)+]; AltName:
Full=NAD(P)H-dependent glycerol-3-phosphate dehydrogenase
Length=327

 Score = 28.1 bits (61),  Expect = 3.4, Method: Composition-based stats.
 Identities = 13/35 (37%), Positives = 20/35 (57%), Gaps = 0/35 (0%)
 Frame = -3

Query  124  TLQGPSAVRDVILNCRGFVQSSLAVGFEPTHSVNS  20
            TL GP+ + D+I+ C      +L+ GF+   S NS
Sbjct  234  TLLGPACLGDLIMTCTSLNSRNLSFGFKIGSSNNS  268


>sp|P61749.1|GPDA_WOLPM RecName: Full=Glycerol-3-phosphate dehydrogenase
[NAD(P)+]; AltName:
Full=NAD(P)H-dependent glycerol-3-phosphate dehydrogenase
Length=327

 Score = 26.9 bits (58),  Expect = 6.5, Method: Composition-based stats.
 Identities = 12/34 (35%), Positives = 20/34 (59%), Gaps = 0/34 (0%)
 Frame = -3

Query  124  TLQGPSAVRDVILNCRGFVQSSLAVGFEPTHSVN  23
            TL GP+ + D+I+ C      +L+ GF+  +S N
Sbjct  234  TLLGPACLGDLIMTCTSLNSRNLSFGFKIGNSDN  267


>sp|C0R3K2.1|GPDA_WOLWR RecName: Full=Glycerol-3-phosphate dehydrogenase
[NAD(P)+]; AltName:
Full=NAD(P)H-dependent glycerol-3-phosphate dehydrogenase
Length=327

 Score = 26.9 bits (58),  Expect = 6.6, Method: Composition-based stats.
 Identities = 12/34 (35%), Positives = 20/34 (59%), Gaps = 0/34 (0%)
 Frame = -3

Query  124  TLQGPSAVRDVILNCRGFVQSSLAVGFEPTHSVN  23
            TL GP+ + D+I+ C      +L+ GF+  +S N
Sbjct  234  TLLGPACLGDLIMTCTSLNSRNLSFGFKIGNSDN  267
```

```
Query= 21

Length=136


                                                                  Score     E
Sequences producing significant alignments:                      (Bits)  Value


sp|Q29293.1|RL3_PIG  RecName: Full=60S ribosomal protein L3        26.6    8.2

ALIGNMENTS


>sp|Q29293.1|RL3_PIG RecName: Full=60S ribosomal protein L3
Length=130

 Score = 26.6 bits (57),  Expect = 8.2, Method: Compositional matrix adjust.
 Identities
 = 10/19 (53%), Positives = 13/19 (68%), Gaps = 0/19 (0%)
 Frame = +2

Query  8    LEDSAVNRMGRFESYGEVV  64
 L D ++N +GRF  YGEV
Sbjct  106  LSDKSINPLGRFVHYGEVT  124


Query= 22

Length=144

 Score     E
Sequences producing significant alignments:                      (Bits)  Value

sp|P49327.3|FAS_HUMAN  RecName: Full=Fatty acid synthase; Incl...  28.9    2.4

ALIGNMENTS
>sp|P49327.3|FAS_HUMAN RecName: Full=Fatty acid synthase; Includes: RecName:
Full=[Acyl-carrier-protein]
S-acetyltransferase; Includes: RecName:
Full=[Acyl-carrier-protein] S-malonyltransferase; Includes:
RecName: Full=3-oxoacyl-[acyl-carrier-protein] synthase; Includes:
RecName: Full=3-oxoacyl-[acyl-carrier-protein] reductase;
Includes: RecName: Full=3-hydroxyacyl-[acyl-carrier-protein]
dehydratase; Includes: RecName: Full=Enoyl-[acyl-carrier-protein]
reductase; Includes: RecName: Full=Oleoyl-[acyl-carrier-protein]
hydrolase
Length=2511

 Score = 28.9 bits (63),  Expect = 2.4, Method: Compositional matrix adjust.
 Identities = 12/27 (44%), Positives = 17/27 (63%), Gaps = 0/27 (0%)
 Frame = -3

Query  142   FTLQGPSAVRDVILNRWLRSIWYGPAH  62
 +TLQ  + V DV+++RWLR    G  H
Sbjct  1067  YTLQDKAQVADVVVSRWLRVTVAGGVH  1093
```

```
Query= 23

Length=149


                                                                      Score     E
Sequences producing significant alignments:                          (Bits)  Value

sp|Q03396.1|L_SV5WR  RecName: Full=RNA-directed RNA polymerase...  28.9    2.4

ALIGNMENTS
>sp|Q03396.1|L_SV5WR RecName: Full=RNA-directed RNA polymerase L; Short=Protein
L;
AltName: Full=Large structural protein; AltName: Full=Replicase;
AltName: Full=Transcriptase; Includes: RecName: Full=RNA-directed
RNA polymerase; Includes: RecName: Full=mRNA (guanine-N(7)-)-methyltransferase;
Includes: RecName: Full=mRNA
guanylyltransferase
Length=2255

 Score = 28.9 bits (63),  Expect = 2.4, Method: Composition-based stats.
 Identities = 15/29 (52%), Positives = 17/29 (59%), Gaps = 3/29 (10%)
 Frame = -3

Query  132  PGPADRRILLN---SDGFDPSGTAQPTTS  55
            P P +RR+LLN    D FDPS   Q  TS
Sbjct  507  PNPFNRRLLLNFLGDDKFDPSVELQYVTS  535


Query= 24

Length=122


                                                                      Score     E
Sequences producing significant alignments:                          (Bits)  Value

sp|Q29293.1|RL3_PIG  RecName: Full=60S ribosomal protein L3        26.2    8.5

ALIGNMENTS
>sp|Q29293.1|RL3_PIG RecName: Full=60S ribosomal protein L3
Length=130

 Score = 26.2 bits (56),  Expect = 8.5, Method: Composition-based stats.
 Identities = 10/18 (56%), Positives = 13/18 (72%), Gaps = 0/18 (0%)
 Frame = +2

Query  8    LEDSAVNRMGRFESYGEV  61
            L D ++N +GRF  YGEV
Sbjct  106  LSDKSINPLGRFVHYGEV  123


Query= 25

Length=136


                                                                      Score     E
Sequences producing significant alignments:                          (Bits)  Value


sp|Q29293.1|RL3_PIG  RecName: Full=60S ribosomal protein L3        26.9    5.8
```

```
ALIGNMENTS

>sp|Q29293.1|RL3_PIG RecName: Full=60S ribosomal protein L3
Length=130

 Score = 26.9 bits (58),  Expect = 5.8, Method: Compositional matrix adjust.
 Identities = 10/19 (53%), Positives = 13/19 (68%), Gaps = 0/19 (0%)
 Frame = +2

Query  8    LEDSAVNRMGRFESYGEVV  64
            L D ++N +GRF  YGEV
Sbjct  106  LSDKSINPLGRFVHYGEVT  124



Query= 26

Length=143


                                                                  Score     E
Sequences producing significant alignments:                      (Bits)  Value

sp|B7VIS0.1|GLND_VIBSL  RecName: Full=[Protein-PII] uridylyltr...  27.7    5.1

ALIGNMENTS
>sp|B7VIS0.1|GLND_VIBSL RecName: Full=[Protein-PII] uridylyltransferase;
Short=PII uridylyl-transferase;
AltName: Full=UTase; AltName: Full=Uridylyl-removing
enzyme
Length=873

 Score = 27.7 bits (60),  Expect = 5.1, Method: Composition-based stats.
 Identities = 16/37 (43%), Positives = 19/37 (51%), Gaps = 2/37 (5%)
 Frame = +3

Query  6    SWKIRLLTEWVGSNPTAKLMRRHLHCGVTATDKFRHN  116
            SWK  LL E   S  T + +RR L   V   D+ RHN
Sbjct  591  SWKRTLLAELFHS--TQRALRRGLENPVDVRDRIRHN  625





Query= 27

Length=133


                                                                  Score     E
Sequences producing significant alignments:                      (Bits)  Value

sp|A9B5T5.1|SYK_HERA2  RecName: Full=Lysine--tRNA ligase; AltN...  29.3    1.5

ALIGNMENTS
>sp|A9B5T5.1|SYK_HERA2 RecName: Full=Lysine--tRNA ligase; AltName: Full=Lysyl-
tRNA synthetase;
Short=LysRS
Length=492

 Score = 29.3 bits (64),  Expect = 1.5, Method: Composition-based stats.
 Identities = 20/43 (47%), Positives = 25/43 (58%), Gaps = 8/43 (19%)
```

```
 Frame = -2

Query  126   ESNDL-----GKLCLNLSEGVEPVLARVPERRFTLQGPSAVRD  13
             E NDL     GKL    + G+EP  ARVP+R  TL   +AVR+
Sbjct  2     ELNDLQQTRYGKLQALQAAGIEPYPARVPQRTHTL---TAVRE  41


Query= 28

Length=124


                                                                    Score     E
Sequences producing significant alignments:                        (Bits)  Value

sp|B3PIT8.1|MNMG_CELJU  RecName: Full=tRNA uridine 5-carboxyme...  32.0    0.18

ALIGNMENTS
>sp|B3PIT8.1|MNMG_CELJU RecName: Full=tRNA uridine 5-carboxymethylaminomethyl
modification
enzyme MnmG; AltName: Full=Glucose-inhibited division
protein A
Length=638

 Score = 32.0 bits (71),  Expect = 0.18, Method: Composition-based stats.
 Identities = 15/31 (48%), Positives = 20/31 (65%), Gaps = 0/31 (0%)
 Frame = +1

Query  31   SLKGEPSSGEYACPQVRVESLRTGWTERIPD  123
            SLKGEP +GE A  QV +E+   G+ +R  D
Sbjct  525  SLKGEPLAGEAAAEQVEIEAKYAGYIDRQQD  555



Query= 29

Length=134


                                                                    Score     E
Sequences producing significant alignments:                        (Bits)  Value


sp|B2RKK1.1|LEPA_PORG3  RecName: Full=Elongation factor 4; Sho...  28.1    3.4
sp|Q7MV56.1|LEPA_PORGI  RecName: Full=Elongation factor 4; Sho...  27.7    4.2


ALIGNMENTS

>sp|B2RKK1.1|LEPA_PORG3 RecName: Full=Elongation factor 4; Short=EF-4; AltName:
Full=Ribosomal
back-translocase LepA
Length=595

 Score = 28.1 bits (61),  Expect = 3.4, Method: Composition-based stats.
 Identities = 12/37 (32%), Positives = 20/37 (54%), Gaps = 3/37 (8%)
 Frame = -2

Query  124  DPSGTAQPTTAERIERPW---QVMSELIWVHPSRTFC  23
            +PSG  +PT  + IE P+    V++   ++ P  T C
Sbjct  385  NPSGLPEPTLIDHIEEPFIRASVITNTAYIGPIMTLC  421


>sp|Q7MV56.1|LEPA_PORGI RecName: Full=Elongation factor 4; Short=EF-4; AltName:
```

```
Full=Ribosomal
back-translocase LepA
Length=595

 Score = 27.7 bits (60),  Expect = 4.2, Method: Composition-based stats.
 Identities = 12/37 (32%), Positives = 20/37 (54%), Gaps = 3/37 (8%)
 Frame = -2

Query  124  DPSGTAQPTTAERIERPW---QVMSELIWVHPSRTFC  23
            +PSG  +PT  + IE P+    V++   ++ P  T C
Sbjct  385  NPSGLLEPTLIDHIEEPFIRASVITNTAYIGPIMTLC  421




Query= 30

Length=124


                                                                 Score     E
Sequences producing significant alignments:                     (Bits)  Value

sp|B1JKQ0.1|PYRF_YERPY  RecName: Full=Orotidine 5'-phosphate d...  28.5    2.2
sp|P58644.1|PYRF_YERPE  RecName: Full=Orotidine 5'-phosphate d...  28.5    2.2
sp|Q66AI1.1|PYRF_YERPS  RecName: Full=Orotidine 5'-phosphate d...  28.1    2.3
sp|A8FVN0.1|PYRF_SHESH  RecName: Full=Orotidine 5'-phosphate d...  28.1    2.8
sp|B4RDU2.1|FMT_PHEZH  RecName: Full=Methionyl-tRNA formyltran...  27.3    4.6
sp|A4WAY2.1|PYRF_ENT38  RecName: Full=Orotidine 5'-phosphate d...  26.6    7.6
sp|A1JM26.1|PYRF_YERE8  RecName: Full=Orotidine 5'-phosphate d...  26.6    8.9

ALIGNMENTS
>sp|B1JKQ0.1|PYRF_YERPY RecName: Full=Orotidine 5'-phosphate decarboxylase;
AltName:
Full=OMP decarboxylase; Short=OMPDCase; Short=OMPdecase
Length=245

 Score = 28.5 bits (62),  Expect = 2.2, Method: Composition-based stats.
 Identities = 13/36 (36%), Positives = 18/36 (50%), Gaps = 0/36 (0%)
 Frame = -2

Query  117  RDSTRTCGQAYSPELCRGFVQSSRFTLQGPSAVRDV  10
            +D+         SP+ CR  V    FTL GP  +RD+
Sbjct  26   KDAALAFADQVSPQDCRLKVGKEMFTLYGPELIRDL  61


>sp|P58644.1|PYRF_YERPE RecName: Full=Orotidine 5'-phosphate decarboxylase;
AltName:
Full=OMP decarboxylase; Short=OMPDCase; Short=OMPdecase
 sp|Q1CJ05.1|PYRF_YERPN RecName: Full=Orotidine 5'-phosphate decarboxylase;
AltName:
Full=OMP decarboxylase; Short=OMPDCase; Short=OMPdecase
 sp|Q1C7L9.1|PYRF_YERPA RecName: Full=Orotidine 5'-phosphate decarboxylase;
AltName:
Full=OMP decarboxylase; Short=OMPDCase; Short=OMPdecase
 sp|A4TJ45.1|PYRF_YERPP RecName: Full=Orotidine 5'-phosphate decarboxylase;
AltName:
Full=OMP decarboxylase; Short=OMPDCase; Short=OMPdecase
 sp|A9R8T1.1|PYRF_YERPG RecName: Full=Orotidine 5'-phosphate decarboxylase;
AltName:
Full=OMP decarboxylase; Short=OMPDCase; Short=OMPdecase
Length=245
```

```
 Score = 28.5 bits (62),  Expect = 2.2, Method: Composition-based stats.
 Identities = 13/36 (36%), Positives = 18/36 (50%), Gaps = 0/36 (0%)
 Frame = -2

Query  117  RDSTRTCGQAYSPELCRGFVQSSRFTLQGPSAVRDV  10
            +D+        SP+ CR  V    FTL GP  +RD+
Sbjct  26   KDAALAFADQVSPQDCRLKVGKEMFTLYGPELIRDL  61


>sp|Q66AI1.1|PYRF_YERPS RecName: Full=Orotidine 5'-phosphate decarboxylase;
AltName:
Full=OMP decarboxylase; Short=OMPDCase; Short=OMPdecase
 sp|A7FI10.1|PYRF_YERP3 RecName: Full=Orotidine 5'-phosphate decarboxylase;
AltName:
Full=OMP decarboxylase; Short=OMPDCase; Short=OMPdecase
 sp|B2K4I4.1|PYRF_YERPB RecName: Full=Orotidine 5'-phosphate decarboxylase;
AltName:
Full=OMP decarboxylase; Short=OMPDCase; Short=OMPdecase
Length=245

 Score = 28.1 bits (61),  Expect = 2.3, Method: Composition-based stats.
 Identities = 13/36 (36%), Positives = 18/36 (50%), Gaps = 0/36 (0%)
 Frame = -2

Query  117  RDSTRTCGQAYSPELCRGFVQSSRFTLQGPSAVRDV  10
            +D+        SP+ CR  V    FTL GP  +RD+
Sbjct  26   KDAALAFADQVSPQDCRLKVGKEMFTLYGPELIRDL  61


>sp|A8FVN0.1|PYRF_SHESH RecName: Full=Orotidine 5'-phosphate decarboxylase;
AltName:
Full=OMP decarboxylase; Short=OMPDCase; Short=OMPdecase
Length=234

 Score = 28.1 bits (61),  Expect = 2.8, Method: Composition-based stats.
 Identities = 11/24 (46%), Positives = 15/24 (63%), Gaps = 0/24 (0%)
 Frame = -2

Query  81  PELCRGFVQSSRFTLQGPSAVRDV  10
           P++CR  V    FTL GP  V+D+
Sbjct  27  PQMCRLKVGKEMFTLFGPELVKDI  50


>sp|B4RDU2.1|FMT_PHEZH RecName: Full=Methionyl-tRNA formyltransferase
Length=308

 Score
 = 27.3 bits (59),  Expect = 4.6, Method: Composition-based stats.
 Identities = 14/34 (41%), Positives = 20/34 (59%), Gaps = 0/34 (0%)
 Frame = -3

Query  113  TQPGPADRRILLNSVGDSFSPAGSPFKDLLLLGT  12
 T+ GP    + LL++  D+  PAG    D LL+GT
Sbjct  239  TEKGPVRVKALLSAFEDAEGPAGETLDDRLLVGT  272


>sp|Q11GT5.1|DNLJ_MESSB RecName: Full=DNA ligase; AltName:
Full=Polydeoxyribonucleotide
synthase [NAD(+)]
Length=703

 Score = 27.3 bits (59),  Expect = 6.5, Method: Composition-based stats.
 Identities = 11/18 (61%), Positives = 14/18 (78%), Gaps = 0/18 (0%)
 Frame = +2
```

```
Query  68   RQSSGEYACPQVRVESLR  121
            R+ SGE+ACP  R+E LR
Sbjct  442  RRCSGEFACPFQRIEHLR  459


>sp|A4WAY2.1|PYRF_ENT38 RecName: Full=Orotidine 5'-phosphate decarboxylase;
AltName:
Full=OMP decarboxylase; Short=OMPDCase; Short=OMPdecase
Length=246

 Score = 26.6 bits (57),  Expect = 7.6, Method: Compositional matrix adjust.
 Identities = 15/36 (42%), Positives = 16/36 (44%), Gaps = 0/36 (0%)
 Frame = -2

Query  117  RDSTRTCGQAYSPELCRGFVQSSRFTLQGPSAVRDV  10
            RDS          P  CR  V    FTL GP  VRD+
Sbjct  26   RDSALAFVDLIDPRDCRLKVGKEMFTLFGPQIVRDL  61


>sp|A1JM26.1|PYRF_YERE8 RecName: Full=Orotidine 5'-phosphate decarboxylase;
AltName:
Full=OMP decarboxylase; Short=OMPDCase; Short=OMPdecase
Length=246

 Score = 26.6 bits (57),  Expect = 8.9, Method: Composition-based stats.
 Identities = 13/36 (36%), Positives = 17/36 (47%), Gaps = 0/36 (0%)
 Frame = -2

Query  117  RDSTRTCGQAYSPELCRGFVQSSRFTLQGPSAVRDV  10
            +D+         +P  CR  V    FTL GP  VRD+
Sbjct  26   KDAALAFADRVNPRDCRLKVGKEMFTLYGPQLVRDL  61


Query= 31

Length=119


                                                                  Score     E
Sequences producing significant alignments:                      (Bits)  Value

sp|Q9UMX2.1|OAZ3_HUMAN  RecName: Full=Ornithine decarboxylase ...  27.7    2.9
sp|Q9CP62.1|MTNN_PASMU  RecName: Full=5'-methylthioadenosine/S...  27.7    3.5

ALIGNMENTS
>sp|Q9UMX2.1|OAZ3_HUMAN RecName: Full=Ornithine decarboxylase antizyme 3;
Short=AZ3;
Short=ODC-Az 3
Length=187

 Score = 27.7 bits (60),  Expect = 2.9, Method: Compositional matrix adjust.
 Identities = 12/28 (43%), Positives = 15/28 (54%), Gaps = 0/28 (0%)
 Frame = -2

Query  118  QNESNDLGKLCLNLSVSGIRSVQPVHPS  35
            QN+ ND G L    S  G   V+P HP+
Sbjct  134  QNDRNDRGALLRAFSYMGFEVVRPDHPA  161


>sp|Q9CP62.1|MTNN_PASMU RecName: Full=5'-methylthioadenosine/S-
adenosylhomocysteine nucleosidase;
Short=MTA/SAH nucleosidase; Short=MTAN; AltName:
Full=5'-methylthioadenosine nucleosidase; Short=MTA nucleosidase;
```

```
AltName: Full=S-adenosylhomocysteine nucleosidase; Short=AdoHcy
nucleosidase; Short=SAH nucleosidase; Short=SRH
nucleosidase
Length=229

 Score = 27.7 bits (60),  Expect = 3.5, Method: Composition-based stats.
 Identities = 14/33 (42%), Positives = 19/33 (58%), Gaps = 2/33 (6%)
 Frame = -1

Query  119  AERI--ERPWQVMSELICVGDSFSPAGSPFKDL  27
            AE+I  ++  QV   LIC GDSF   G+P   +
Sbjct  129  AEKIAVQQQQQVKRGLICSGDSFIQGGTPLAQI  161




Query= 32

Length=144


                                                                  Score     E
Sequences producing significant alignments:                      (Bits)  Value

sp|Q0V6M5.1|LCPS_PHANO  RecName: Full=Bifunctional lycopene cy...  27.3    7.3

ALIGNMENTS
>sp|Q0V6M5.1|LCPS_PHANO RecName: Full=Bifunctional lycopene cyclase/phytoene
synthase;
Includes: RecName: Full=Lycopene beta-cyclase; AltName: Full=Lycopene
cyclase; Includes: RecName: Full=Phytoene synthase
Length=585

 Score = 27.3 bits (59),  Expect = 7.3, Method: Composition-based stats.
 Identities = 13/29 (45%), Positives = 18/29 (62%), Gaps = 1/29 (3%)
 Frame = +2

Query  41   VNQWWAGPYQMDRSHLL*LEDSAVNRMGR  127
            +N  W  PY+ DR+ L  L+D AV R+ R
Sbjct  268  INALWVSPYKYDRARLAGLQD-AVLRLKR  295




Query= 33

Length=148


                                                                  Score     E
Sequences producing significant alignments:                      (Bits)  Value

sp|A8ABM5.1|EF1A_IGNH4  RecName: Full=Elongation factor 1-alph...  29.6    1.1

ALIGNMENTS
>sp|A8ABM5.1|EF1A_IGNH4 RecName: Full=Elongation factor 1-alpha; Short=EF-1-
alpha; AltName:
Full=Elongation factor Tu; Short=EF-Tu
Length=442

 Score = 29.6 bits (65),  Expect = 1.1, Method: Composition-based stats.
 Identities = 17/46 (37%), Positives = 25/46 (54%), Gaps = 5/46 (11%)
 Frame = -3
```

```
Query  146  AVGFEP-----THSVNSRIFQLQQGVEPVLARVPERRSGGTQPGPA  24
            AVG+ P     T SV SRI +++Q ++P   +V E      +PG A
Sbjct  344  AVGYTPVIHAHTASVASRIIEIKQKIDPRTGKVIEENPSFLKPGDA  389



Query= 34

Length=116


                                                                 Score     E
Sequences producing significant alignments:                      (Bits)  Value

sp|Q9FY99.2|G6PD2_ARATH  RecName: Full=Glucose-6-phosphate 1-d...  27.3    5.4
 sp|B2SX19.1|GPDA_BURPP  RecName: Full=Glycerol-3-phosphate deh...  26.6    8.5

ALIGNMENTS
>sp|Q9FY99.2|G6PD2_ARATH RecName: Full=Glucose-6-phosphate 1-dehydrogenase 2,
chloroplastic;
Short=G6PD2; Short=G6PDH2; Flags: Precursor
Length=596

 Score = 27.3 bits (59),  Expect = 5.4, Method: Compositional matrix adjust.
 Identities = 13/25 (52%), Positives = 14/25 (56%), Gaps = 0/25 (0%)
 Frame = -2

Query  106  SFSPARGSNPY*PEFLSGGPEGLNP  32
            SFSP  G       FLS  P+GLNP
Sbjct  20   SFSPVNGDRHRSLSFLSASPQGLNP  44



>sp|B2SX19.1|GPDA_BURPP RecName: Full=Glycerol-3-phosphate dehydrogenase
[NAD(P)+]; AltName:
Full=NAD(P)H-dependent glycerol-3-phosphate dehydrogenase
Length=332

 Score = 26.6 bits (57),  Expect = 8.5, Method: Compositional matrix adjust.
 Identities = 14/36 (39%), Positives = 20/36 (56%), Gaps = 2/36 (6%)
 Frame = -1

Query  116  CRGFVQSSQGV--EPVLARVPERRSGGTQPGPADRR  15
            C+GF   SQ +  + V A +PE +S G   GP+  R
Sbjct  107  CKGFEADSQLMPHQVVAAELPEHKSNGVLSGPSFAR  142


>sp|Q9I2N4.1|MODC_PSEAE RecName: Full=Molybdenum import ATP-binding protein ModC
Length=361

 Score = 26.6 bits (57),  Expect = 10.0, Method: Compositional matrix adjust.
 Identities = 11/18 (61%), Positives = 14/18 (78%), Gaps = 0/18 (0%)
 Frame = -1

Query  89   GVEPVLARVPERRSGGTQ  36
            G+E +L R+PER SGG Q
Sbjct  123  GIEHLLERMPERLSGGEQ  140
```

```
Query= 35

Length=138


                                                                      Score     E
Sequences producing significant alignments:                          (Bits)  Value


sp|A7HIL1.1|Y4379_ANADF  RecName: Full=Probable RNA methyltran...  26.9    7.6
sp|Q8ZIN5.1|KHSE_YERPE  RecName: Full=Homoserine kinase; Short...  26.9    7.9
sp|Q2NVZ5.1|KHSE_SODGM  RecName: Full=Homoserine kinase; Short...  26.9    8.5
sp|A1JJC7.1|KHSE_YERE8  RecName: Full=Homoserine kinase; Short...  26.6    8.9

ALIGNMENTS
>sp|A7HIL1.1|Y4379_ANADF RecName: Full=Probable RNA methyltransferase
Anae109_4379
Length=336

 Score = 26.9 bits (58),  Expect = 7.6, Method: Compositional matrix adjust.
 Identities = 11/24 (46%), Positives = 17/24 (71%), Gaps = 0/24 (0%)
 Frame = -1

Query  102  GAERIERPWQVMSELIWVRRDSTR  31
            G  R  R W+++++L+ VR DSTR
Sbjct  120  GLARSLRAWEIVAQLLHVRADSTR  143




>sp|Q8ZIN5.1|KHSE_YERPE RecName: Full=Homoserine kinase; Short=HK; Short=HSK
 sp|Q66ET8.1|KHSE_YERPS RecName: Full=Homoserine kinase; Short=HK; Short=HSK
 sp|Q1C0K9.1|KHSE_YERPA RecName: Full=Homoserine kinase; Short=HK; Short=HSK
 sp|Q1CMW6.1|KHSE_YERPN RecName: Full=Homoserine kinase; Short=HK; Short=HSK
 sp|A4TQG9.1|KHSE_YERPP RecName: Full=Homoserine kinase; Short=HK; Short=HSK
 sp|A7FMF2.1|KHSE_YERP3 RecName: Full=Homoserine kinase; Short=HK; Short=HSK
 sp|B2K3L2.1|KHSE_YERPB RecName: Full=Homoserine kinase; Short=HK; Short=HSK
 sp|A9R025.1|KHSE_YERPG RecName: Full=Homoserine kinase; Short=HK; Short=HSK
 sp|B1JL13.1|KHSE_YERPY RecName: Full=Homoserine kinase; Short=HK; Short=HSK
Length=309

 Score = 26.9 bits (58),  Expect = 7.9, Method: Composition-based stats.
 Identities = 13/31 (42%), Positives = 19/31 (61%), Gaps = 0/31 (0%)
 Frame = -2

Query  131  RTRISPSS*AAQNESNDLGKLCLNLSGSGGT  39
            RT++ P   AA+  + D+G L   +SGSG T
Sbjct  235  RTQLLPGFAAARQAAQDIGALACGISGSGPT  265


>sp|Q2NVZ5.1|KHSE_SODGM RecName: Full=Homoserine kinase; Short=HK; Short=HSK
Length=310

 Score = 26.9 bits (58),  Expect = 8.5, Method: Composition-based stats.
 Identities = 15/39 (38%), Positives = 20/39 (51%), Gaps = 0/39 (0%)
 Frame = -2

Query  131  RTRISPSS*AAQNESNDLGKLCLNLSGSGGTQPGPADRR  15
            RTR+ P    A+    D+G L   +SGSG T     DR+
Sbjct  235  RTRLLPGFADARQAVGDIGALACGISGSGPTLFAICDRQ  273
```

```
>sp|A1JJC7.1|KHSE_YERE8 RecName: Full=Homoserine kinase; Short=HK; Short=HSK
Length=309

 Score = 26.6 bits (57),  Expect = 8.9, Method: Composition-based stats.
 Identities = 13/31 (42%), Positives = 19/31 (61%), Gaps = 0/31 (0%)
 Frame = -2

Query  131  RTRISPSS*AAQNESNDLGKLCLNLSGSGGT  39
            RT++ P   AA+  + D+G L   +SGSG T
Sbjct  235  RTQLLPGFAAARQAAQDIGALACGISGSGPT  265



Query= 36

Length=146


                                                                  Score     E
Sequences producing significant alignments:                      (Bits)  Value

sp|Q9SW95.2|SYNC2_ARATH  RecName: Full=Asparagine--tRNA ligase...  29.3    1.9

ALIGNMENTS
>sp|Q9SW95.2|SYNC2_ARATH RecName: Full=Asparagine--tRNA ligase, cytoplasmic 2;
AltName:
Full=Asparaginyl-tRNA synthetase 2; Short=AsnRS 2
Length=638

 Score = 29.3 bits (64),  Expect = 1.9, Method: Composition-based stats.
 Identities = 12/40 (30%), Positives = 17/40 (43%), Gaps = 0/40 (0%)
 Frame = -3

Query  132  GPSAVRDVILNAERIERPWQVMSELIWVRRDSTRTCGQAY  13
            GP  + D I NA  +   W V +E+ +   D    C   Y
Sbjct  401  GPRFIADKIDNARHLAEKWNVETEMAFAELDDAMDCADEY  440




Query  23   PQVRVESLRTGWTERIPDSLGSRP  94
            PQVR+    T W++R P +L S P
Sbjct  833  PQVRIAPTVTTWSDRTPTALPSHP  856




Query= 37

Length=139


                                                                  Score     E
Sequences producing significant alignments:                      (Bits)  Value

sp|P49327.3|FAS_HUMAN  RecName: Full=Fatty acid synthase; Incl...  29.6    1.2

ALIGNMENTS
>sp|P49327.3|FAS_HUMAN RecName: Full=Fatty acid synthase; Includes: RecName:
Full=[Acyl-carrier-protein]
S-acetyltransferase; Includes: RecName:
Full=[Acyl-carrier-protein] S-malonyltransferase; Includes:
RecName: Full=3-oxoacyl-[acyl-carrier-protein] synthase; Includes:
```

```
RecName: Full=3-oxoacyl-[acyl-carrier-protein] reductase;
Includes: RecName: Full=3-hydroxyacyl-[acyl-carrier-protein]
dehydratase; Includes: RecName: Full=Enoyl-[acyl-carrier-protein]
reductase; Includes: RecName: Full=Oleoyl-[acyl-carrier-protein]
hydrolase
Length=2511

 Score = 29.6 bits (65),  Expect = 1.2, Method: Compositional matrix adjust.
 Identities = 14/37 (38%), Positives = 20/37 (54%), Gaps = 0/37 (0%)
 Frame = -3

Query  137   FTLQGPSAVRDVILNRWLRSIWYGPAHHWSGGTQPGP  27
             +TLQ   + V DV+++RWLR    G  H     T+  P
Sbjct  1067  YTLQDKAQVADVVVSRWLRVTVAGGVHISGLHTESAP  1103




Query= 38

Length=117


                                                                      Score     E
Sequences producing significant alignments:                          (Bits)  Value


sp|Q0V6M5.1|LCPS_PHANO  RecName: Full=Bifunctional lycopene cy...  27.3    6.5


ALIGNMENTS


>sp|Q0V6M5.1|LCPS_PHANO RecName: Full=Bifunctional lycopene cyclase/phytoene
synthase;
Includes: RecName: Full=Lycopene beta-cyclase; AltName: Full=Lycopene
cyclase; Includes: RecName: Full=Phytoene synthase
Length=585

 Score = 27.3 bits (59),  Expect = 6.5, Method: Compositional matrix adjust.
 Identities = 9/18 (50%), Positives = 13/18 (72%), Gaps = 0/18 (0%)
 Frame = +1

Query  58   WAGPYQMDRSHLAGLNES  111
            W  PY+ DR+ LAGL ++
Sbjct  272  WVSPYKYDRARLAGLQDA  289




Query= 39

Length=116


                                                                      Score     E
Sequences producing significant alignments:                          (Bits)  Value

sp|A1K5J1.1|SYE_AZOSB  RecName: Full=Glutamate--tRNA ligase; A...  28.1    3.0


ALIGNMENTS
```

```
>sp|A1K5J1.1|SYE_AZOSB RecName: Full=Glutamate--tRNA ligase; AltName:
Full=Glutamyl-tRNA
synthetase; Short=GluRS
Length=467

 Score = 28.1 bits (61),  Expect = 3.0, Method: Compositional matrix adjust.
 Identities = 15/28 (54%), Positives = 18/28 (64%), Gaps = 1/28 (4%)
 Frame = +3

Query  27   RSGLSP-SGPAHETTLALRSDR*AGLNE  107
            R G+ P +GPA E  +AL  DR A LNE
Sbjct  336  RRGVDPEAGPALEQVVALYKDRSANLNE  363




Query= 40

Length=126


                                                                  Score     E
Sequences producing significant alignments:                      (Bits)  Value

sp|Q2KA45.1|PUR5_RHIEC  RecName: Full=Phosphoribosylformylglyc...  26.6    8.6


ALIGNMENTS
>sp|Q2KA45.1|PUR5_RHIEC RecName: Full=Phosphoribosylformylglycinamidine cyclo-
ligase;
AltName: Full=AIR synthase; AltName: Full=AIRS; AltName: Full=Phosphoribosyl-
aminoimidazole
synthetase
Length=357

 Score = 26.6 bits (57),  Expect = 8.6, Method: Composition-based stats.
 Identities = 15/39 (38%), Positives = 21/39 (54%), Gaps = 1/39 (3%)
 Frame = +3

Query  9    FRHNLPRSFDSFCAAQ-ELGLIRVRPPSGGLGRTRWIEA  122
            F  N+PR      AA+ +LG ++V P    L RT  +EA
Sbjct  259  FPENIPRVLPEHLAAEIDLGAVKVPPVFSWLARTGGVEA  297




Query= 41

Length=143


                                                                  Score     E
Sequences producing significant alignments:                      (Bits)  Value

sp|Q8GXG1.2|ASPG2_ARATH  RecName: Full=Probable isoaspartyl pe...  27.7    5.2


ALIGNMENTS
>sp|Q8GXG1.2|ASPG2_ARATH RecName: Full=Probable isoaspartyl peptidase/L-
asparaginase 2;
AltName: Full=L-asparagine amidohydrolase 2; Contains: RecName:
Full=Isoaspartyl peptidase/L-asparaginase 2 subunit alpha;
Contains: RecName: Full=Isoaspartyl peptidase/L-asparaginase
2 subunit beta; Flags: Precursor
```

```
Length=325

 Score = 27.7 bits (60),  Expect = 5.2, Method: Composition-based stats.
 Identities = 13/24 (54%), Positives = 14/24 (58%), Gaps = 0/24 (0%)
 Frame = -2

Query  76  G*QPNLPATAQNESNDLGKLCLNL  5
           G  PNLPA  Q E+  L   CLNL
Sbjct  13  GIDPNLPAERQEEAKQLLTRCLNL  36



Query= 42

Length=151


                                                                      Score     E
Sequences producing significant alignments:                          (Bits)  Value

sp|Q8GXG1.2|ASPG2_ARATH  RecName: Full=Probable isoaspartyl pe...  28.5    2.8

ALIGNMENTS
>sp|Q8GXG1.2|ASPG2_ARATH RecName: Full=Probable isoaspartyl peptidase/L-
asparaginase 2;
AltName: Full=L-asparagine amidohydrolase 2; Contains: RecName:
Full=Isoaspartyl peptidase/L-asparaginase 2 subunit alpha;
Contains: RecName: Full=Isoaspartyl peptidase/L-asparaginase
2 subunit beta; Flags: Precursor
Length=325

 Score = 28.5 bits (62),  Expect = 2.8, Method: Composition-based stats.
 Identities = 13/24 (54%), Positives = 14/24 (58%), Gaps = 0/24 (0%)
 Frame = -1

Query  76  G*QPNLPATAQNESNDLGKLCLNL  5
           G  PNLPA  Q E+  L   CLNL
Sbjct  13  GIDPNLPAERQEEAKQLLTRCLNL  36



Query= 43

Length=129


                                                                      Score     E
Sequences producing significant alignments:                          (Bits)  Value

sp|Q8GXG1.2|ASPG2_ARATH  RecName: Full=Probable isoaspartyl pe...  28.5    2.2

ALIGNMENTS
>sp|Q8GXG1.2|ASPG2_ARATH RecName: Full=Probable isoaspartyl peptidase/L-
asparaginase 2;
AltName: Full=L-asparagine amidohydrolase 2; Contains: RecName:
Full=Isoaspartyl peptidase/L-asparaginase 2 subunit alpha;
Contains: RecName: Full=Isoaspartyl peptidase/L-asparaginase
2 subunit beta; Flags: Precursor
Length=325

 Score = 28.5 bits (62),  Expect = 2.2, Method: Composition-based stats.
 Identities = 13/24 (54%), Positives = 14/24 (58%), Gaps = 0/24 (0%)
 Frame = -3
```

```
Query  76  G*QPNLPATAQNESNDLGKLCLNL  5
           G  PNLPA  Q E+  L   CLNL
Sbjct  13  GIDPNLPAERQEEAKQLLTRCLNL  36
```

Query= 44

Length=151

```
                                                                      Score     E
Sequences producing significant alignments:                          (Bits)  Value


sp|A3QDC9.1|CDD_SHELP  RecName: Full=Cytidine deaminase; AltNa...  27.3    6.3
```

ALIGNMENTS

>sp|A3QDC9.1|CDD_SHELP RecName: Full=Cytidine deaminase; AltName: Full=Cytidine aminohydrolase;
Short=CDA
Length=296

```
 Score = 27.3 bits (59),  Expect = 6.3, Method: Compositional matrix adjust.
 Identities = 13/42 (31%), Positives = 22/42 (52%), Gaps = 0/42 (0%)
 Frame = -3

Query  128  HSVNSRIFQLQRFTLQGPSAVRDVILNAERIERPWQVMSELI  3
            HSV++    +    L G + + D+I+NA       Q M+EL+
Sbjct  98   HSVHAEQSAISHAWLSGETGIEDIIVNASPCGHCRQFMNELV  139
```

Query= 45

Length=146

```
                                                                      Score     E
Sequences producing significant alignments:                          (Bits)  Value


sp|B3PIT8.1|MNMG_CELJU  RecName: Full=tRNA uridine 5-carboxyme...  27.3    7.0
```

ALIGNMENTS

>sp|B3PIT8.1|MNMG_CELJU RecName: Full=tRNA uridine 5-carboxymethylaminomethyl modification
enzyme MnmG; AltName: Full=Glucose-inhibited division
protein A
Length=638

```
 Score = 27.3 bits (59),  Expect = 7.0, Method: Composition-based stats.
 Identities = 12/20 (60%), Positives = 15/20 (75%), Gaps = 0/20 (0%)
 Frame = +3

Query  78   SLKGEPSSGEYACPQVRVES  137
            SLKGEP +GE A  QV +E+
Sbjct  525  SLKGEPLAGEAAAEQVEIEA  544
```

```
 Score = 27.3 bits (59),  Expect = 6.0, Method: Composition-based stats.
 Identities = 12/34 (35%), Positives = 23/34 (68%), Gaps = 3/34 (9%)
 Frame = -1

Query  134  RWLRSIWYGPAHHWFTLQGPSAVRDVILNAERIE  33
            RW +S + G A H+FT+  P   R+++L+ +++E
Sbjct  14   RWDQSTFLGRARHFFTVTDP---RNLLLSGKQLE  44



Query= 46

Length=138


                                                                      Score     E
Sequences producing significant alignments:                          (Bits)  Value

sp|A1BGY2.1|KTHY_CHLPD  RecName: Full=Thymidylate kinase; AltN...  28.1    2.6
sp|Q8FLM0.1|SYL_COREF  RecName: Full=Leucine--tRNA ligase; Alt...  27.3    6.4
sp|B3EDD6.1|KTHY_CHLL2  RecName: Full=Thymidylate kinase; AltN...  26.9    6.8
sp|Q9A842.1|DNLJ_CAUCR  RecName: Full=DNA ligase; AltName: Ful...  26.9    9.9

ALIGNMENTS
>sp|A1BGY2.1|KTHY_CHLPD RecName: Full=Thymidylate kinase; AltName: Full=dTMP
kinase
Length=219

 Score = 28.1 bits (61),  Expect = 2.6, Method: Composition-based stats.
 Identities = 18/42 (43%), Positives = 24/42 (57%), Gaps = 4/42 (10%)
 Frame = -3

Query  136  GVEPVLARVPERRSGGTQPGPADRRILLNSQNESNDLGKLCL  11
            GVE V  R P    GGT+     R ILL S++E + +G+L L
Sbjct  28   GVESVTLREP----GGTEVAEKIRSILLESRHEISAVGELLL  65


>sp|Q8FLM0.1|SYL_COREF RecName: Full=Leucine--tRNA ligase; AltName: Full=Leucyl-
tRNA
synthetase; Short=LeuRS
Length=957

 Score = 27.3 bits (59),  Expect = 6.4, Method: Composition-based stats.
 Identities = 11/22 (50%), Positives = 14/22 (64%), Gaps = 0/22 (0%)
 Frame = -2

Query  86  STRTCGQAYSPELAERIERPWQ  21
           S ++    Y+PELA  IER WQ
Sbjct  11  SAQSLAYRYTPELANAIEREWQ  32


>sp|B3EDD6.1|KTHY_CHLL2 RecName: Full=Thymidylate kinase; AltName: Full=dTMP
kinase
Length=219

 Score = 26.9 bits (58),  Expect = 6.8, Method: Composition-based stats.
 Identities = 13/31 (42%), Positives = 20/31 (65%), Gaps = 0/31 (0%)
 Frame = -3

Query  103  RRSGGTQPGPADRRILLNSQNESNDLGKLCL  11
            R  GGT+     R+ILL S++E + +G+L L
Sbjct  35   REPGGTEVAEKIRQILLESRHEISPIGELLL  65
```

```
Query= 47

Length=156


                                                                      Score     E
Sequences producing significant alignments:                          (Bits)  Value

sp|A3MXT2.1|HEM12_PYRCJ  RecName: Full=Glutamyl-tRNA reductase...  30.8    0.53
sp|B1I165.1|KTHY_DESAP  RecName: Full=Thymidylate kinase; AltN...  27.7    5.0
sp|Q8FLM0.1|SYL_COREF  RecName: Full=Leucine--tRNA ligase; Alt...  27.3    7.1
sp|B3EDD6.1|KTHY_CHLL2  RecName: Full=Thymidylate kinase; AltN...  26.9    9.1

ALIGNMENTS
>sp|A3MXT2.1|HEM12_PYRCJ RecName: Full=Glutamyl-tRNA reductase 2; Short=GluTR 2
Length=391

 Score = 30.8 bits (68),  Expect = 0.53, Method: Compositional matrix adjust.
 Identities = 12/37 (32%), Positives = 20/37 (54%), Gaps = 0/37 (0%)
 Frame = -2

Query  119  PNLPATAVRRDSTRTCGQAYSPELAERIERPWQVMSE  9
            PNLP   V+ +  R   + Y+ E A  +E+ W ++ E
Sbjct  270  PNLPVAVVKIEDLRELAEEYNRERAGEVEKAWAIVDE  306



>sp|B1I165.1|KTHY_DESAP RecName: Full=Thymidylate kinase; AltName: Full=dTMP
kinase
Length=221

 Score = 27.7 bits (60),  Expect = 5.0, Method: Composition-based stats.
 Identities = 12/28 (43%), Positives = 18/28 (64%), Gaps = 0/28 (0%)
 Frame = -3

Query  124  NSRIFQLQRSGGTQPGPADRRILLNSQN  41
            N R+    +  GGT+ G A RR+LL++ N
Sbjct  32   NHRVLVTREPGGTRIGEAVRRVLLDTGN  59


>sp|Q8FLM0.1|SYL_COREF RecName: Full=Leucine--tRNA ligase; AltName: Full=Leucyl-
tRNA
synthetase; Short=LeuRS
Length=957

 Score = 27.3 bits (59),  Expect = 7.1, Method: Composition-based stats.
 Identities = 11/22 (50%), Positives = 14/22 (64%), Gaps = 0/22 (0%)
 Frame = -2

Query  86  STRTCGQAYSPELAERIERPWQ  21
           S ++    Y+PELA  IER WQ
Sbjct  11  SAQSLAYRYTPELANAIEREWQ  32


>sp|B3EDD6.1|KTHY_CHLL2 RecName: Full=Thymidylate kinase; AltName: Full=dTMP
kinase
Length=219

 Score = 26.9 bits (58),  Expect = 9.1, Method: Composition-based stats.
 Identities = 13/32 (41%), Positives = 21/32 (66%), Gaps = 0/32 (0%)
 Frame = -3
```

```
Query  106   LQRSGGTQPGPADRRILLNSQNESNDLGKLCL  11
             L+   GGT+      R+ILL S++E + +G+L L
Sbjct  34    LREPGGTEVAEKIRQILLESRHEISPIGELLL  65


Query= 48

Length=139


                                                                  Score     E
Sequences producing significant alignments:                      (Bits)  Value

sp|Q8FLM0.1|SYL_COREF  RecName: Full=Leucine--tRNA ligase; Alt...  27.7    5.9
sp|Q966C6.3|RL7A_CAEEL  RecName: Full=60S ribosomal protein L7a    26.9    8.3
sp|Q04462.2|SYVC_RAT  RecName: Full=Valine--tRNA ligase; AltNa...  26.9    9.0

ALIGNMENTS

>sp|Q8FLM0.1|SYL_COREF RecName: Full=Leucine--tRNA ligase; AltName: Full=Leucyl-
tRNA
synthetase; Short=LeuRS
Length=957

 Score = 27.7 bits (60),  Expect = 5.9, Method: Composition-based stats.
 Identities = 11/22 (50%), Positives = 14/22 (64%), Gaps = 0/22 (0%)
 Frame = -3

Query  86  STRTCGQAYSPELAERIERPWQ  21
           S ++    Y+PELA  IER WQ
Sbjct  11  SAQSLAYRYTPELANAIEREWQ  32


>sp|Q966C6.3|RL7A_CAEEL RecName: Full=60S ribosomal protein L7a
Length=265

 Score = 26.9 bits (58),  Expect = 8.3, Method: Composition-based stats.
 Identities = 11/29 (38%), Positives = 15/29 (52%), Gaps = 0/29 (0%)
 Frame = -1

Query  103  HWSGGTQPGPADRRILLNSQNESNDLGKL  17
            HW GG      +D + L    +  + DLGKL
Sbjct  237  HWGGGVMSAKSDAKKLKIERARARDLGKL  265


>sp|Q04462.2|SYVC_RAT RecName: Full=Valine--tRNA ligase; AltName: Full=Valyl-
tRNA synthetase;
Short=ValRS
Length=1264

 Score = 26.9 bits (58),  Expect = 9.0, Method: Compositional matrix adjust.
 Identities = 11/30 (37%), Positives = 17/30 (57%), Gaps = 0/30 (0%)
 Frame = -3

Query  113  PSPPLVRRDSTRTCGQAYSPELAERIERPW  24
            P+PP  ++D + T   +YSP+  E    PW
Sbjct  283  PTPPGEKKDVSGTMPDSYSPQYVEAAWYPW  312
```

```
Query= 49

Length=138


                                                                      Score     E
Sequences producing significant alignments:                          (Bits)  Value

sp|P16084.1|BGLS_BUTFI  RecName: Full=Beta-glucosidase A; AltN...  30.0    0.99

ALIGNMENTS
>sp|P16084.1|BGLS_BUTFI RecName: Full=Beta-glucosidase A; AltName: Full=Beta-D-
glucoside
glucohydrolase; AltName: Full=Cellobiase; AltName: Full=Gentiobiase
Length=830

 Score = 30.0 bits (66),  Expect = 0.99, Method: Composition-based stats.
 Identities = 15/33 (45%), Positives = 17/33 (52%), Gaps = 0/33 (0%)
 Frame = -3

Query  124  QCKCRLMSWSGGTQPGPADRRILLNSQNESNDL  26
            Q    LM+W GG + G A  RILL   N S  L
Sbjct  227  QISSVLMAWQGGIEGGLAAARILLGKVNPSGKL  259


Query= 50

Length=129


                                                                      Score     E
Sequences producing significant alignments:                          (Bits)  Value


sp|Q8DLB7.1|PYRD_THEEB  RecName: Full=Dihydroorotate dehydroge...  26.6    8.7

ALIGNMENTS


>sp|Q8DLB7.1|PYRD_THEEB RecName: Full=Dihydroorotate dehydrogenase (quinone);
AltName:
Full=DHOdehase; Short=DHOD; Short=DHODase; AltName: Full=Dihydroorotate
oxidase
Length=364

 Score = 26.6 bits (57),  Expect = 8.7, Method: Composition-based stats.
 Identities = 10/29 (34%), Positives = 15/29 (52%), Gaps = 0/29 (0%)
 Frame = +3

Query  21   LPRSFDSFCGWTERIPDSCSWKIRLLTEW  107
            L + F S CGW  +    C+W  + L +W
Sbjct  25   LHQQFLSLCGWLNQDRSLCTWLRQQLQQW  53



Query= 51

Length=134


                                                                      Score     E
Sequences producing significant alignments:                          (Bits)  Value

sp|P49327.3|FAS_HUMAN  RecName: Full=Fatty acid synthase; Incl...  28.5    2.7
```

```
ALIGNMENTS
>sp|P49327.3|FAS_HUMAN RecName: Full=Fatty acid synthase; Includes: RecName:
Full=[Acyl-carrier-protein]
S-acetyltransferase; Includes: RecName:
Full=[Acyl-carrier-protein] S-malonyltransferase; Includes:
RecName: Full=3-oxoacyl-[acyl-carrier-protein] synthase; Includes:
RecName: Full=3-oxoacyl-[acyl-carrier-protein] reductase;
Includes: RecName: Full=3-hydroxyacyl-[acyl-carrier-protein]
dehydratase; Includes: RecName: Full=Enoyl-[acyl-carrier-protein]
reductase; Includes: RecName: Full=Oleoyl-[acyl-carrier-protein]
hydrolase
Length=2511

 Score = 28.5 bits (62),  Expect = 2.7, Method: Compositional matrix adjust.
 Identities = 12/27 (44%), Positives = 17/27 (63%), Gaps = 0/27 (0%)
 Frame = -3

Query  132   FTLQGPSAVRDVILNRWLRSIWYGPAH  52
             +TLQ   + V DV+++RWLR    G  H
Sbjct  1067  YTLQDKAQVADVVVSRWLRVTVAGGVH  1093



Query= 52

Length=139


                                                                  Score     E
Sequences producing significant alignments:                       (Bits)  Value

sp|Q165T7.1|G6PI_ROSDO  RecName: Full=Glucose-6-phosphate isom...  28.9    1.9

ALIGNMENTS

>sp|Q165T7.1|G6PI_ROSDO RecName: Full=Glucose-6-phosphate isomerase; Short=GPI;
AltName:
Full=Phosphoglucose isomerase; Short=PGI; AltName: Full=Phosphohexose
isomerase; Short=PHI
Length=530

 Score = 28.9 bits (63),  Expect = 1.9, Method: Composition-based stats.
 Identities = 13/29 (45%), Positives = 17/29 (59%), Gaps = 0/29 (0%)
 Frame = +3

Query  24   PRSFDSFCSGGLGRTRWIEAI*VQENTPV  110
            PR+FD+F  GG    R  +A   +EN PV
Sbjct  267  PRAFDAFLRGGQAMDRHFQAADWRENLPV  2


Query= 53

Length=112


                                                                  Score     E
Sequences producing significant alignments:                       (Bits)  Value

sp|Q0V6M5.1|LCPS_PHANO  RecName: Full=Bifunctional lycopene cy...  27.3    6.2

ALIGNMENTS
>sp|Q0V6M5.1|LCPS_PHANO RecName: Full=Bifunctional lycopene cyclase/phytoene
synthase;
```

```
Includes: RecName: Full=Lycopene beta-cyclase; AltName: Full=Lycopene
cyclase; Includes: RecName: Full=Phytoene synthase
Length=585

 Score = 27.3 bits (59),  Expect = 6.2, Method: Compositional matrix adjust.
 Identities = 9/18 (50%), Positives = 13/18 (72%), Gaps = 0/18 (0%)
 Frame = +2

Query  53   WAGPYQMDRSHLAGLNES  106
            W  PY+ DR+ LAGL ++
Sbjct  272  WVSPYKYDRARLAGLQDA
  289



Query= 54

Length=116


 Score     E
Sequences producing significant alignments:                       (Bits)  Value

sp|Q1QX56.2|PLSX_CHRSD  RecName: Full=Phosphate acyltransferas...  26.9    6.7

ALIGNMENTS
>sp|Q1QX56.2|PLSX_CHRSD RecName: Full=Phosphate acyltransferase; AltName:
Full=Acyl-ACP
phosphotransacylase; AltName: Full=Acyl-[acyl-carrier-protein]--phosphate
acyltransferase; AltName: Full=Phosphate-acyl-ACP
acyltransferase
Length=379

 Score = 26.9 bits (58),  Expect = 6.7, Method: Compositional matrix adjust.
 Identities = 12/32 (38%), Positives = 20/32 (63%), Gaps = 0/32 (0%)
 Frame = -1

Query  104  LNPDLRTGVFS*TQGVEPVLARVPERLSGIRS  9
 ++PDL   ++   + +E  L  +P+RLSG RS
Sbjct  29   MHPDLEIQMYGPREALEDELETLPKRLSGYRS  60




Query= 55

Length=129


 Score     E
Sequences producing significant alignments:                       (Bits)  Value

sp|Q2T1H1.2|GPDA_BURTA  RecName: Full=Glycerol-3-phosphate deh...  29.3    1.4
sp|Q5S2C3.2|PIR_ARATH  RecName: Full=Protein PIR; AltName: Ful...  26.9    8.7

ALIGNMENTS
>sp|Q2T1H1.2|GPDA_BURTA RecName: Full=Glycerol-3-phosphate dehydrogenase
[NAD(P)+]; AltName:
Full=NAD(P)H-dependent glycerol-3-phosphate dehydrogenase
Length=332

 Score = 29.3 bits (64),  Expect = 1.4, Method: Composition-based stats.
 Identities = 15/35 (43%), Positives = 19/35 (54%), Gaps = 6/35 (17%)
 Frame = -3
```

```
Query  121  LQGPSAVRDVILNSP*DSNLPIRLTAESSSYSCRG  17
            L GPS  R+V         LP+ LT  S+S +CRG
Sbjct  135  LSGPSFAREVA------QGLPVALTVASASAACRG  163


>sp|Q5S2C3.2|PIR_ARATH RecName: Full=Protein PIR; AltName: Full=PIR of plants;
AltName:
Full=Protein KLUNKER; Short=AtSRA1; AltName: Full=Protein
PIROGI
Length=1282

 Score = 26.9 bits (58),  Expect = 8.7, Method: Composition-based stats.
 Identities = 15/38 (39%), Positives = 19/38 (50%), Gaps = 1/38 (3%)
 Frame = +1

Query  4    WTERIPDSCSWKIRLLTEWVGSNPTASLGSRP*QQKVL  117
            WT RI + C+WK        G  P AS GS    +KV+
Sbjct  413  WTARIWEQCAWKFSRPCRDAGETPEAS-GSYSDYEKVV  449


Query= 56

Length=134


                                                                      Score     E
Sequences producing significant alignments:                          (Bits)  Value

sp|Q29293.1|RL3_PIG  RecName: Full=60S ribosomal protein L3        27.3    4.0

ALIGNMENTS
>sp|Q29293.1|RL3_PIG RecName: Full=60S ribosomal protein L3
Length=130

 Score = 27.3 bits (59),  Expect = 4.0, Method: Compositional matrix adjust.
 Identities = 9/23 (39%), Positives = 15/23 (65%), Gaps = 0/23 (0%)
 Frame = +3

Query  33   LEDSAVNRMGRFESYGESSGEYA  101
            L D ++N +GRF  YGE + ++
Sbjct  106  LSDKSINPLGRFVHYGEVTNDFV  128



Query= 57

Length=122


                                                                      Score     E
Sequences producing significant alignments:                          (Bits)  Value

sp|Q29293.1|RL3_PIG  RecName: Full=60S ribosomal protein L3        26.2    7.5

ALIGNMENTS
>sp|Q29293.1|RL3_PIG RecName: Full=60S ribosomal protein L3
Length=130

 Score = 26.2 bits (56),  Expect = 7.5, Method: Composition-based stats.
 Identities = 10/18 (56%), Positives = 13/18 (72%), Gaps = 0/18 (0%)
 Frame = +3

Query  33   LEDSAVNRMGRFESYGEV  86
```

```
             L D ++N +GRF  YGEV
Sbjct  106  LSDKSINPLGRFVHYGEV  123



Query= 58

Length=124


                                                                  Score     E
Sequences producing significant alignments:                      (Bits)  Value


sp|B3PIT8.1|MNMG_CELJU  RecName: Full=tRNA uridine 5-carboxyme...  27.3    6.1

ALIGNMENTS

>sp|B3PIT8.1|MNMG_CELJU RecName: Full=tRNA uridine 5-carboxymethylaminomethyl
modification
enzyme MnmG; AltName: Full=Glucose-inhibited division
protein A
Length=638

 Score = 27.3 bits (59),  Expect = 6.1, Method: Composition-based stats.
 Identities = 12/20 (60%), Positives = 15/20 (75%), Gaps = 0/20 (0%)
 Frame = +2

Query  56   SLKGEPSSGEYACPQVRVES  115
            SLKGEP +GE A  QV +E+
Sbjct  525  SLKGEPLAGEAAAEQVEIEA  544


Query= 59

Length=119


                                                                  Score     E
Sequences producing significant alignments:                      (Bits)  Value

sp|B1KDM8.1|PYRD_SHEWM  RecName: Full=Dihydroorotate dehydroge...  27.7    3.3

ALIGNMENTS
>sp|B1KDM8.1|PYRD_SHEWM RecName: Full=Dihydroorotate dehydrogenase (quinone);
AltName:
Full=DHOdehase; Short=DHOD; Short=DHODase; AltName: Full=Dihydroorotate
oxidase
Length=340

 Score = 27.7 bits (60),  Expect = 3.3, Method: Composition-based stats.
 Identities = 10/19 (53%), Positives = 13/19 (68%), Gaps = 0/19 (0%)
 Frame = -2

Query  76   SGFTLQGPSAVRDVILNCR  20
            SGF  QGP  ++D+I  CR
Sbjct  319  SGFIYQGPKLIKDIIEACR  337
```

```
Query= 60

Length=124


                                                                      Score     E
Sequences producing significant alignments:                          (Bits)  Value


sp|B1ZGG2.1|MNMG_METPB  RecName: Full=tRNA uridine 5-carboxyme...  28.1    3.8
sp|Q8Y003.1|RGMG_RALSO  RecName: Full=Putative ribose/galactos...  26.9    8.2

ALIGNMENTS

>sp|B1ZGG2.1|MNMG_METPB RecName: Full=tRNA uridine 5-carboxymethylaminomethyl
modification
enzyme MnmG; AltName: Full=Glucose-inhibited division
protein A
Length=624

 Score = 28.1 bits (61),  Expect = 3.8, Method: Compositional matrix adjust.
 Identities = 16/40 (40%), Positives = 24/40
 (60%), Gaps = 3/40 (8%)
 Frame = +1

Query  7    TERIPDRVQEN---TPVRRSGLSPSGPR*VQT*LAKVVRF  117
 T+R+ D ++EN   +P+   G+S  GPR   +   KVVRF
Sbjct  248  TQRVHDLIRENLHRSPMYSGGISSRGPRYCPSIEDKVVRF  287


>sp|Q8Y003.1|RGMG_RALSO RecName: Full=Putative ribose/galactose/methyl
galactoside import
ATP-binding protein
Length=518

 Score = 26.9 bits (58),  Expect = 8.2, Method: Compositional matrix adjust.
 Identities = 11/29 (38%), Positives = 16/29 (55%), Gaps = 0/29 (0%)
 Frame = -1

Query  106  PWQVMSELIWVRRDSTRTCGQAYSPELCR  20
 P+  ++E IW+RR+    CG     EL R
Sbjct  116  PYMTVAENIWIRREPRNRCGLVDHAELRR  144




Query= 61

Length=116


 Score     E
Sequences producing significant alignments:                          (Bits)  Value

sp|P16084.1|BGLS_BUTFI  RecName: Full=Beta-glucosidase A; AltN...  28.1    3.0

ALIGNMENTS
>sp|P16084.1|BGLS_BUTFI RecName: Full=Beta-glucosidase A; AltName: Full=Beta-D-
glucoside
glucohydrolase; AltName: Full=Cellobiase; AltName: Full=Gentiobiase
Length=830
```

```
 Score = 28.1 bits (61),  Expect = 3.0, Method: Composition-based stats.
 Identities = 14/30 (47%), Positives = 16/30 (53%), Gaps = 0/30 (0%)
 Frame = -3

Query  102  QCKCRLMSWSGGTQPGPADRRILLNSVGDS  13
            Q    LM+W GG + G A  RILL  V  S
Sbjct  227  QISSVLMAWQGGIEGGLAAARILLGKVNPS  256




Query= 62

Length=112


                                                                      Score     E
Sequences producing significant alignments:                          (Bits)  Value

sp|P49327.3|FAS_HUMAN  RecName: Full=Fatty acid synthase; Incl...  28.9    1.8
sp|O27121.1|PGK_METTH  RecName: Full=Phosphoglycerate kinase       27.7    3.7

ALIGNMENTS
>sp|P49327.3|FAS_HUMAN RecName: Full=Fatty acid synthase; Includes: RecName: 
Full=[Acyl-carrier-protein] 
S-acetyltransferase; Includes: RecName: 
Full=[Acyl-carrier-protein] S-malonyltransferase; Includes: 
RecName: Full=3-oxoacyl-[acyl-carrier-protein] synthase; Includes: 
RecName: Full=3-oxoacyl-[acyl-carrier-protein] reductase; 
Includes: RecName: Full=3-hydroxyacyl-[acyl-carrier-protein] 
dehydratase; Includes: RecName: Full=Enoyl-[acyl-carrier-protein] 
reductase; Includes: RecName: Full=Oleoyl-[acyl-carrier-protein] 
hydrolase
Length=2511

 Score = 28.9 bits (63),  Expect = 1.8, Method: Compositional matrix adjust.
 Identities = 14/34 (41%), Positives = 22/34 (65%), Gaps = 1/34 (3%)
 Frame = -3

Query  110   FTLQGPSAVRDVILNRWLRSIWYGPAHHLSGIRS  9
             +TLQ   + V DV+++RWLR    G   H+SG+ +
Sbjct  1067  YTLQDKAQVADVVVSRWLRVTVAG-GVHISGLHT  1099


>sp|O27121.1|PGK_METTH RecName: Full=Phosphoglycerate kinase
Length=411

 Score = 27.7 bits (60),  Expect = 3.7, Method: Compositional matrix adjust.
 Identities = 13/30 (43%), Positives = 18/30 (60%), Gaps = 4/30 (13%)
 Frame = -2

Query  78   RDPKQMASIHLVRPSPPL----VGDSFSPA  1
            RDP++ A  HLVR   PL    + D+F+ A
Sbjct  134  RDPEEQAETHLVRKLAPLLDYFINDAFAAA  163
```

```
Query= 63

Length=136


                                                                      Score     E
Sequences producing significant alignments:                          (Bits)  Value

sp|Q0RRP6.1|RS13_FRAAA  RecName: Full=30S ribosomal protein S13    26.2    9.1

ALIGNMENTS
>sp|Q0RRP6.1|RS13_FRAAA RecName: Full=30S ribosomal protein S13
Length=126

 Score = 26.2 bits (56),  Expect = 9.1, Method: Compositional matrix adjust.
 Identities = 13/40 (33%), Positives = 20/40 (50%), Gaps = 0/40 (0%)
 Frame = +1

Query  7    GLGRTRWIEAICRSGTRANTGSTPCCSWKIRLLTEWVGSN  126
            G+GRTR  E +  +G   +T        +I  L EW+ +N
Sbjct  24   GIGRTRSRETLAATGVNPDTRVRDLSEDEIVRLREWIDAN  63


Query= 64

Length=131


                                                                      Score     E
Sequences producing significant alignments:                          (Bits)  Value

sp|A6X1X9.1|PLSX_OCHA4  RecName: Full=Phosphate acyltransferas...  27.7    4.5
sp|A5VPV7.1|PLSX_BRUO2  RecName: Full=Phosphate acyltransferas...  27.3    5.5
sp|Q57DX5.1|PLSX_BRUAB  RecName: Full=Phosphate acyltransferas...  27.3    5.5
sp|Q8YGH8.1|PLSX_BRUME  RecName: Full=Phosphate acyltransferas...  27.3    5.5
sp|Q8G1E1.1|PLSX_BRUSU  RecName: Full=Phosphate acyltransferas...  27.3    5.5

ALIGNMENTS
>sp|A6X1X9.1|PLSX_OCHA4 RecName: Full=Phosphate acyltransferase; AltName:
Full=Acyl-ACP
phosphotransacylase; AltName: Full=Acyl-[acyl-carrier-protein]--phosphate
acyltransferase; AltName: Full=Phosphate-acyl-ACP
acyltransferase
Length=352

 Score = 27.7 bits (60),  Expect = 4.5, Method: Composition-based stats.
 Identities = 14/29 (48%), Positives = 18/29 (62%), Gaps = 0/29 (0%)
 Frame = -1

Query  116  NPDLRTGVFS*TQGVEPVLARVPERQMAS  30
            +PD+R   F     VEPVLAR P+ + AS
Sbjct  29   HPDIRFIFFGLPAQVEPVLARYPKLKAAS  57


>sp|A5VPV7.1|PLSX_BRUO2 RecName: Full=Phosphate acyltransferase; AltName:
Full=Acyl-ACP
phosphotransacylase; AltName: Full=Acyl-[acyl-carrier-protein]--phosphate
acyltransferase; AltName: Full=Phosphate-acyl-ACP
acyltransferase
Length=346
```

```
 Score = 27.3 bits (59),  Expect = 5.5, Method: Composition-based stats.
 Identities = 14/29 (48%), Positives = 18/29 (62%), Gaps = 0/29 (0%)
 Frame = -1

Query  116   NPDLRTGVFS*TQGVEPVLARVPERQMAS  30
             +PD+R   F     VEPVLAR P+ + AS
Sbjct  29    HPDIRFIFFGLPAQVEPVLARYPKLKEAS  57


>sp|Q57DX5.1|PLSX_BRUAB RecName: Full=Phosphate acyltransferase; AltName: 
Full=Acyl-ACP 
phosphotransacylase; AltName: Full=Acyl-[acyl-carrier-protein]--phosphate 
acyltransferase; AltName: Full=Phosphate-acyl-ACP 
acyltransferase
 sp|Q2YNC0.1|PLSX_BRUA2 RecName: Full=Phosphate acyltransferase; AltName: 
Full=Acyl-ACP 
phosphotransacylase; AltName: Full=Acyl-[acyl-carrier-protein]--phosphate 
acyltransferase; AltName: Full=Phosphate-acyl-ACP 
acyltransferase
 sp|A9MAF5.1|PLSX_BRUC2 RecName: Full=Phosphate acyltransferase; AltName: 
Full=Acyl-ACP 
phosphotransacylase; AltName: Full=Acyl-[acyl-carrier-protein]--phosphate 
acyltransferase; AltName: Full=Phosphate-acyl-ACP 
acyltransferase
 sp|B0CLA1.1|PLSX_BRUSI RecName: Full=Phosphate acyltransferase; AltName: 
Full=Acyl-ACP 
phosphotransacylase; AltName: Full=Acyl-[acyl-carrier-protein]--phosphate 
acyltransferase; AltName: Full=Phosphate-acyl-ACP 
acyltransferase
 sp|B2S523.1|PLSX_BRUA1 RecName: Full=Phosphate acyltransferase; AltName: 
Full=Acyl-ACP 
phosphotransacylase; AltName: Full=Acyl-[acyl-carrier-protein]--phosphate 
acyltransferase; AltName: Full=Phosphate-acyl-ACP 
acyltransferase
Length=346

 Score = 27.3 bits (59),  Expect = 5.5, Method: Composition-based stats.
 Identities = 14/29 (48%), Positives = 18/29 (62%), Gaps = 0/29 (0%)
 Frame = -1

Query  116   NPDLRTGVFS*TQGVEPVLARVPERQMAS  30
             +PD+R   F     VEPVLAR P+ + AS
Sbjct  29    HPDIRFIFFGLPAQVEPVLARYPKLKEAS  57


>sp|Q8YGH8.1|PLSX_BRUME RecName: Full=Phosphate acyltransferase; AltName: 
Full=Acyl-ACP 
phosphotransacylase; AltName: Full=Acyl-[acyl-carrier-protein]--phosphate 
acyltransferase; AltName: Full=Phosphate-acyl-ACP 
acyltransferase
 sp|C0RIB2.1|PLSX_BRUMB RecName: Full=Phosphate acyltransferase; AltName: 
Full=Acyl-ACP 
phosphotransacylase; AltName: Full=Acyl-[acyl-carrier-protein]--phosphate 
acyltransferase; AltName: Full=Phosphate-acyl-ACP 
acyltransferase
Length=346

 Score = 27.3 bits (59),  Expect = 5.5, Method: Composition-based stats.
 Identities = 14/29 (48%), Positives = 18/29 (62%), Gaps = 0/29 (0%)
 Frame = -1

Query  116   NPDLRTGVFS*TQGVEPVLARVPERQMAS  30
             +PD+R   F     VEPVLAR P+ + AS
Sbjct  29    HPDIRFIFFGLPAQVEPVLARYPKLKEAS  57
```

```
>sp|Q8G1E1.1|PLSX_BRUSU RecName: Full=Phosphate acyltransferase; AltName:
Full=Acyl-ACP
phosphotransacylase; AltName: Full=Acyl-[acyl-carrier-protein]--phosphate
acyltransferase; AltName: Full=Phosphate-acyl-ACP
acyltransferase
Length=346

 Score = 27.3 bits (59),  Expect = 5.5, Method: Composition-based stats.
 Identities = 14/29 (48%), Positives = 18/29 (62%), Gaps = 0/29 (0%)
 Frame = -1

Query  116  NPDLRTGVFS*TQGVEPVLARVPERQMAS  30
            +PD+R   F     VEPVLAR P+ + AS
Sbjct  29   HPDIRFIFFGLPAQVEPVLARYPKLKEAS  57


Query= 65

Length=126


                                                                  Score     E
Sequences producing significant alignments:                      (Bits)  Value

sp|P78605.1|OTC_TRAHI  RecName: Full=Ornithine carbamoyltransf...  28.1    3.6
sp|Q4R5T4.1|RNF32_MACFA  RecName: Full=RING finger protein 32      26.9    7.3

ALIGNMENTS
>sp|P78605.1|OTC_TRAHI RecName: Full=Ornithine carbamoyltransferase,
mitochondrial;
AltName: Full=Ornithine transcarbamylase; Short=OTCase; Flags:
Precursor
Length=375

 Score = 28.1 bits (61),  Expect = 3.6, Method: Composition-based stats.
 Identities = 14/29 (48%), Positives = 16/29 (55%), Gaps = 5/29 (17%)
 Frame = +1

Query  31   EAICRSGTRANTGSTPSDKFRHNLPRSFD  117
            EA+CR G     G+ P  KF H LPR  D
Sbjct  301  EALCREG-----GANPDWKFMHCLPRKQD  324


>sp|Q4R5T4.1|RNF32_MACFA RecName: Full=RING finger protein 32
Length=362

 Score = 26.9 bits (58),  Expect = 7.3, Method: Composition-based stats.
 Identities = 12/17 (71%), Positives = 14/17 (82%), Gaps = 0/17 (0%)
 Frame = +2

Query  44   AQELGLIRVRPPQISSD  94
            AQ+LGLI   PPQ+SSD
Sbjct  92   AQKLGLIGPPPPQLSSD  108
```

```
Query= 66

Length=144


                                                                      Score     E
Sequences producing significant alignments:                          (Bits)  Value

sp|Q0V6M5.1|LCPS_PHANO  RecName: Full=Bifunctional lycopene cy...  27.3    6.8

ALIGNMENTS
>sp|Q0V6M5.1|LCPS_PHANO RecName: Full=Bifunctional lycopene cyclase/phytoene
synthase;
Includes: RecName: Full=Lycopene beta-cyclase; AltName: Full=Lycopene
cyclase; Includes: RecName: Full=Phytoene synthase
Length=585

 Score = 27.3 bits (59),  Expect = 6.8, Method: Compositional matrix adjust.
 Identities = 18/44 (41%), Positives = 23/44 (52%), Gaps = 3/44 (7%)
 Frame = +3

Query  6    WAGPYQMDRSHLL*LEDSAVNRMGRFESYGEFRITSLTAEGP*R  137
            W  PY+ DR+ L  L+D AV R+ R      F + S T  GP R
Sbjct  272  WVSPYKYDRARLAGLQD-AVLRLKR--KSRSFYLASATFPGPLR  312



Query= 67

Length=122


                                                                      Score     E
Sequences producing significant alignments:                          (Bits)  Value

sp|P45694.2|TKT_BACSU  RecName: Full=Transketolase; Short=TK       28.5    2.8

ALIGNMENTS
>sp|P45694.2|TKT_BACSU RecName: Full=Transketolase; Short=TK
Length=667

 Score = 28.5 bits (62),  Expect = 2.8, Method: Composition-based stats.
 Identities = 13/36 (36%), Positives = 22/36 (61%), Gaps = 1/36 (3%)
 Frame = -1

Query  113  FVQSSLAVGFE-PTHSVNSRIFQLQQMASIHLVRPS  9
            F   S+AVG + PTH    ++  L+ M ++ L+RP+
Sbjct  459  FTHDSIAVGEDGPTHEPVEQLASLRAMPNLSLIRPA  494


Query= 68

Length=136


                                                                      Score     E
Sequences producing significant alignments:                          (Bits)  Value

sp|Q47N76.1|SYFB_THEFY  RecName: Full=Phenylalanine--tRNA liga...  28.5    2.9

ALIGNMENTS
>sp|Q47N76.1|SYFB_THEFY RecName: Full=Phenylalanine--tRNA ligase beta subunit;
AltName:
Full=Phenylalanyl-tRNA synthetase beta subunit; Short=PheRS
```

```
Length=831

 Score = 28.5 bits (62),  Expect = 2.9, Method: Composition-based stats.
 Identities = 15/33 (45%), Positives = 20/33 (61%), Gaps = 1/33 (3%)
 Frame = +1

Query  34   AICCSWKIRLLTEWVGSNPTAKLMRRHLH-CGV  129
            AIC  + +R +T +   + PT   MRR LH CGV
Sbjct  220  AICSRYVLRGVTGFDPAAPTPLWMRRRLHLCGV  252



Query= 69

Length=126


                                                                      Score     E
Sequences producing significant alignments:                          (Bits)  Value

sp|P49327.3|FAS_HUMAN  RecName: Full=Fatty acid synthase; Incl...  29.6    1.2

ALIGNMENTS
>sp|P49327.3|FAS_HUMAN RecName: Full=Fatty acid synthase; Includes: RecName:
Full=[Acyl-carrier-protein]
S-acetyltransferase; Includes: RecName:
Full=[Acyl-carrier-protein] S-malonyltransferase; Includes:
RecName: Full=3-oxoacyl-[acyl-carrier-protein] synthase; Includes:
RecName: Full=3-oxoacyl-[acyl-carrier-protein] reductase;
Includes: RecName: Full=3-hydroxyacyl-[acyl-carrier-protein]
dehydratase; Includes: RecName: Full=Enoyl-[acyl-carrier-protein]
reductase; Includes: RecName: Full=Oleoyl-[acyl-carrier-protein]
hydrolase
Length=2511

 Score = 29.6 bits (65),  Expect = 1.2, Method: Compositional matrix adjust.
 Identities = 12/30 (40%), Positives = 19/30 (63%), Gaps = 0/30 (0%)
 Frame = -3

Query  94    ERRFTLQGPSAVRDVILNRWLRSIWYGPAH  5
             ++ +TLQ  + V DV+++RWLR    G  H
Sbjct  1064  QKLYTLQDKAQVADVVVSRWLRVTVAGGVH  1093


Query= 70

Length=144


                                                                      Score     E
Sequences producing significant alignments:                          (Bits)  Value

sp|P49327.3|FAS_HUMAN  RecName: Full=Fatty acid synthase; Incl...  28.9    2.6

ALIGNMENTS
>sp|P49327.3|FAS_HUMAN RecName: Full=Fatty acid synthase; Includes: RecName:
Full=[Acyl-carrier-protein]
S-acetyltransferase; Includes: RecName:
Full=[Acyl-carrier-protein] S-malonyltransferase; Includes:
RecName: Full=3-oxoacyl-[acyl-carrier-protein] synthase; Includes:
RecName: Full=3-oxoacyl-[acyl-carrier-protein] reductase;
Includes: RecName: Full=3-hydroxyacyl-[acyl-carrier-protein]
dehydratase; Includes: RecName: Full=Enoyl-[acyl-carrier-protein]
reductase; Includes: RecName: Full=Oleoyl-[acyl-carrier-protein]
```

```
Query= 71

Length=139


                                                                      Score     E
Sequences producing significant alignments:                          (Bits)  Value

sp|P49327.3|FAS_HUMAN  RecName: Full=Fatty acid synthase; Incl...  28.5    2.9
sp|B3PIT8.1|MNMG_CELJU  RecName: Full=tRNA uridine 5-carboxyme...  27.3    6.7

ALIGNMENTS
>sp|P49327.3|FAS_HUMAN RecName: Full=Fatty acid synthase; Includes: RecName:
Full=[Acyl-carrier-protein]
S-acetyltransferase; Includes: RecName:
Full=[Acyl-carrier-protein] S-malonyltransferase; Includes:
RecName: Full=3-oxoacyl-[acyl-carrier-protein] synthase; Includes:
RecName: Full=3-oxoacyl-[acyl-carrier-protein] reductase;
Includes: RecName: Full=3-hydroxyacyl-[acyl-carrier-protein]
dehydratase; Includes: RecName: Full=Enoyl-[acyl-carrier-protein]
reductase; Includes: RecName: Full=Oleoyl-[acyl-carrier-protein]
hydrolase
Length=2511

 Score = 28.5 bits (62),  Expect = 2.9, Method: Compositional matrix adjust.
 Identities = 12/27 (44%), Positives = 17/27 (63%), Gaps = 0/27 (0%)
 Frame = -1

Query  85    FTLQGPSAVRDVILNRWLRSIWYGPAH  5
             +TLQ  + V DV+++RWLR    G  H
Sbjct  1067  YTLQDKAQVADVVVSRWLRVTVAGGVH  1093


>sp|B3PIT8.1|MNMG_CELJU RecName: Full=tRNA uridine 5-carboxymethylaminomethyl
modification
enzyme MnmG; AltName: Full=Glucose-inhibited division
protein A
Length=638

 Score = 27.3 bits (59),  Expect = 6.7, Method: Composition-based stats.
 Identities = 12/20 (60%), Positives = 15/20 (75%), Gaps = 0/20 (0%)
 Frame = +2

Query  71   SLKGEPSSGEYACPQVRVES  130
            SLKGEP +GE A  QV +E+
Sbjct  525  SLKGEPLAGEAAAEQVEIEA  544


Query= 72

Length=134


                                                                      Score     E
Sequences producing significant alignments:                          (Bits)  Value

sp|P49327.3|FAS_HUMAN  RecName: Full=Fatty acid synthase; Incl...  29.3    1.8

ALIGNMENTS
>sp|P49327.3|FAS_HUMAN RecName: Full=Fatty acid synthase; Includes: RecName:
Full=[Acyl-carrier-protein]
S-acetyltransferase; Includes: RecName:
Full=[Acyl-carrier-protein] S-malonyltransferase; Includes:
RecName: Full=3-oxoacyl-[acyl-carrier-protein] synthase; Includes:
```

```
RecName: Full=3-oxoacyl-[acyl-carrier-protein] reductase;
Includes: RecName: Full=3-hydroxyacyl-[acyl-carrier-protein]
dehydratase; Includes: RecName: Full=Enoyl-[acyl-carrier-protein]
reductase; Includes: RecName: Full=Oleoyl-[acyl-carrier-protein]
hydrolase
Length=2511

 Score = 29.3 bits (64),  Expect = 1.8, Method: Compositional matrix adjust.
 Identities = 12/27 (44%), Positives = 17/27 (63%), Gaps = 0/27 (0%)
 Frame = -2

Query  85    FTLQGPSAVRDVILNRWLRSIWYGPAH  5
             +TLQ   + V DV+++RWLR    G  H
Sbjct  1067  YTLQDKAQVADVVVSRWLRVTVAGGVH  1093


Query= 73

Length=112


                                                                  Score     E
Sequences producing significant alignments:                       (Bits)  Value

sp|P49327.3|FAS_HUMAN  RecName: Full=Fatty acid synthase; Incl...  28.5    2.5

ALIGNMENTS
>sp|P49327.3|FAS_HUMAN RecName: Full=Fatty acid synthase; Includes: RecName:
Full=[Acyl-carrier-protein]
S-acetyltransferase; Includes: RecName:
Full=[Acyl-carrier-protein] S-malonyltransferase; Includes:
RecName: Full=3-oxoacyl-[acyl-carrier-protein] synthase; Includes:
RecName: Full=3-oxoacyl-[acyl-carrier-protein] reductase;
Includes: RecName: Full=3-hydroxyacyl-[acyl-carrier-protein]
dehydratase; Includes: RecName: Full=Enoyl-[acyl-carrier-protein]
reductase; Includes: RecName: Full=Oleoyl-[acyl-carrier-protein]
hydrolase
Length=2511

 Score = 28.5 bits (62),  Expect = 2.5, Method: Compositional matrix adjust.
 Identities = 12/27 (44%), Positives = 17/27 (63%), Gaps = 0/27 (0%)
 Frame = -1

Query  85    FTLQGPSAVRDVILNRWLRSIWYGPAH  5
             +TLQ   + V DV+++RWLR    G  H
Sbjct  1067  YTLQDKAQVADVVVSRWLRVTVAGGVH  1093


Query= 74

Length=126


                                                                  Score     E
Sequences producing significant alignments:                       (Bits)  Value

sp|P49327.3|FAS_HUMAN  RecName: Full=Fatty acid synthase; Incl...  28.9    2.4

ALIGNMENTS
>sp|P49327.3|FAS_HUMAN RecName: Full=Fatty acid synthase; Includes: RecName:
Full=[Acyl-carrier-protein]
S-acetyltransferase; Includes: RecName:
Full=[Acyl-carrier-protein] S-malonyltransferase; Includes:
RecName: Full=3-oxoacyl-[acyl-carrier-protein] synthase; Includes:
```

```
RecName: Full=3-oxoacyl-[acyl-carrier-protein] reductase;
Includes: RecName: Full=3-hydroxyacyl-[acyl-carrier-protein]
dehydratase; Includes: RecName: Full=Enoyl-[acyl-carrier-protein]
reductase; Includes: RecName: Full=Oleoyl-[acyl-carrier-protein]
hydrolase
Length=2511

 Score = 28.9 bits (63),  Expect = 2.4, Method: Compositional matrix adjust.
 Identities = 12/27 (44%), Positives = 17/27 (63%), Gaps = 0/27 (0%)
 Frame = -3

Query  85    FTLQGPSAVRDVILNRWLRSIWYGPAH  5
             +TLQ  + V DV+++RWLR    G  H
Sbjct  1067  YTLQDKAQVADVVVSRWLRVTVAGGVH  1093



Query= 75

Length=139


                                                                      Score     E
Sequences producing significant alignments:                          (Bits)  Value

sp|Q9BI06.1|R27A1_ENTHI  RecName: Full=60S ribosomal protein L...  31.2    0.17
sp|Q9BH80.1|R27A2_ENTHI  RecName: Full=60S ribosomal protein L...  30.8    0.27
sp|Q9BI14.1|R27A3_ENTHI  RecName: Full=60S ribosomal protein L...  30.0    0.55
sp|Q8Y003.1|RGMG_RALSO  RecName: Full=Putative ribose/galactos...  26.9    8.2

ALIGNMENTS
>sp|Q9BI06.1|R27A1_ENTHI RecName: Full=60S ribosomal protein L27a-1
Length=149

 Score = 31.2 bits (69),  Expect = 0.17, Method: Composition-based stats.
 Identities = 13/41 (32%), Positives = 23/41 (56%), Gaps = 0/41 (0%)
 Frame = +3

Query  12   GPYQMDRSHLSSGEYACPQVRVESLRTQISSDITCQGRSIR  134
            G + M   HL + +Y CP + V+SL + +  DI  Q ++ +
Sbjct  54   GKHGMRVFHLKANKYYCPSINVDSLWSLVGKDIQAQYKNAK  94


>sp|Q9BH80.1|R27A2_ENTHI RecName: Full=60S ribosomal protein L27a-2/4
Length=149

 Score = 30.8 bits (68),  Expect = 0.27, Method: Composition-based stats.
 Identities = 12/41 (29%), Positives = 23/41 (56%), Gaps = 0/41 (0%)
 Frame = +3

Query  12   GPYQMDRSHLSSGEYACPQVRVESLRTQISSDITCQGRSIR  134
            G + M   HL + +Y CP + V+SL + +  D+  Q ++ +
Sbjct  54   GKHGMRVFHLKANKYYCPSINVDSLWSLVGKDVQAQYKNAK  94


>sp|Q9BI14.1|R27A3_ENTHI RecName: Full=60S ribosomal protein L27a-3
Length=149

 Score = 30.0 bits (66),  Expect = 0.55, Method: Composition-based stats.
 Identities = 12/42 (29%), Positives = 24/42 (57%), Gaps = 0/42 (0%)
 Frame = +3

Query  12   GPYQMDRSHLSSGEYACPQVRVESLRTQISSDITCQGRSIRS  137
            G + M   HL + +Y CP + V+SL + +  D+  Q ++ ++
```

```
Sbjct  54   GKHGMRVFHLKANKYYCPSINVDSLWSLVGKDVQEQYKNAKT  95



>sp|Q8Y003.1|RGMG_RALSO RecName: Full=Putative ribose/galactose/methyl
galactoside import
ATP-binding protein
Length=518

 Score = 26.9 bits (58),  Expect = 8.2, Method: Compositional matrix adjust.
 Identities = 11/28 (39%), Positives = 16/28 (57%), Gaps = 0/28 (0%)
 Frame = -1

Query  121  PWQVMSELIWVRRDSTRTCGQAYSPELR  38
            P+  ++E IW+RR+    CG     ELR
Sbjct  116  PYMTVAENIWIRREPRNRCGLVDHAELR  143




Query= 76

Length=131


                                                                  Score     E
Sequences producing significant alignments:                      (Bits)  Value

sp|P16084.1|BGLS_BUTFI  RecName: Full=Beta-glucosidase A; AltN...  28.1    3.9


ALIGNMENTS
>sp|P16084.1|BGLS_BUTFI RecName: Full=Beta-glucosidase A; AltName: Full=Beta-D-
glucoside
glucohydrolase; AltName: Full=Cellobiase; AltName: Full=Gentiobiase
Length=830

 Score = 28.1 bits (61),  Expect = 3.9, Method: Composition-based stats.
 Identities = 16/38 (42%), Positives = 19/38 (50%), Gaps = 2/38 (5%)
 Frame = -3

Query  117  QCKCRLMSWSGGTQPGPADRRILLNSDGFDPSGTAQPT  4
            Q    LM+W GG + G A  RILL     +PSG    T
Sbjct  227  QISSVLMAWQGGIEGGLAAARILLGK--VNPSGKLSDT  262
```

```
Query= 77

Length=104


                                                                      Score     E
Sequences producing significant alignments:                          (Bits)  Value

sp|Q0V6M5.1|LCPS_PHANO  RecName: Full=Bifunctional lycopene cy...  26.9    7.7

ALIGNMENTS
>sp|Q0V6M5.1|LCPS_PHANO RecName: Full=Bifunctional lycopene cyclase/phytoene
synthase;
Includes: RecName: Full=Lycopene beta-cyclase; AltName: Full=Lycopene
cyclase; Includes: RecName: Full=Phytoene synthase
Length=585

 Score = 26.9 bits (58),  Expect = 7.7, Method: Compositional matrix adjust.
 Identities = 9/18 (50%), Positives = 13/18 (72%), Gaps = 0/18 (0%)
 Frame = +3

Query  6    WAGPYQMDRSHLAGLNES  59
            W  PY+ DR+ LAGL ++
Sbjct  272  WVSPYKYDRARLAGLQDA  289



Query= 78

Length=117


                                                                      Score     E
Sequences producing significant alignments:                          (Bits)  Value

sp|Q0V6M5.1|LCPS_PHANO  RecName: Full=Bifunctional lycopene cy...  28.5    2.1
sp|P35341.1|L_SV41  RecName: Full=RNA-directed RNA polymerase ...  28.5    2.3

ALIGNMENTS
>sp|Q0V6M5.1|LCPS_PHANO RecName: Full=Bifunctional lycopene cyclase/phytoene
synthase;
Includes: RecName: Full=Lycopene beta-cyclase; AltName: Full=Lycopene
cyclase; Includes: RecName: Full=Phytoene synthase
Length=585

 Score = 28.5 bits (62),  Expect = 2.1, Method: Compositional matrix adjust.
 Identities = 10/24 (42%), Positives = 15/24 (63%), Gaps = 0/24 (0%)
 Frame = +3

Query  6    WAGPYQMDRSHLAGLNESPTEFRR  77
            W  PY+ DR+ LAGL ++    +R
Sbjct  272  WVSPYKYDRARLAGLQDAVLRLKR  295


>sp|P35341.1|L_SV41 RecName: Full=RNA-directed RNA polymerase L; Short=Protein
L;
AltName: Full=Large structural protein; AltName: Full=Replicase;
AltName: Full=Transcriptase; Includes: RecName: Full=RNA-directed
RNA polymerase; Includes: RecName: Full=mRNA (guanine-N(7)-)-methyltransferase;
Includes: RecName: Full=mRNA
guanylyltransferase
Length=2269

 Score = 28.5 bits (62),  Expect = 2.3, Method: Composition-based stats.
```

```
 Identities = 14/30 (47%), Positives = 19/30 (63%), Gaps = 1/30 (3%)
 Frame = -3

Query  100  PGPADRRILLNSVGD-SFSPARWLRSIWYG  14
            P P +RR+LLN + D SF PA+ L  +  G
Sbjct  509  PNPFNRRLLLNFIEDPSFDPAKELEYVTSG  538




Query= 79

Length=104


                                                                     Score     E
Sequences producing significant alignments:                         (Bits)  Value

sp|Q0V6M5.1|LCPS_PHANO  RecName: Full=Bifunctional lycopene cy...  26.6    9.1

ALIGNMENTS
>sp|Q0V6M5.1|LCPS_PHANO RecName: Full=Bifunctional lycopene cyclase/phytoene
synthase;
Includes: RecName: Full=Lycopene beta-cyclase; AltName: Full=Lycopene
cyclase; Includes: RecName: Full=Phytoene synthase
Length=585

 Score = 26.6 bits (57),  Expect = 9.1, Method: Composition-based stats.
 Identities = 9/18 (50%), Positives = 13/18 (72%), Gaps = 0/18 (0%)
 Frame = +3

Query  6    WAGPYQMDRSHLAGLNES  59
            W  PY+ DR+ LAGL ++
Sbjct  272  WVSPYKYDRARLAGLQDA  289




Query= 80

Length=126


                                                                     Score     E
Sequences producing significant alignments:                         (Bits)  Value

sp|Q6XQH7.2|L_OLVVA  RecName: Full=RNA-directed RNA polymerase...  30.8    0.56

ALIGNMENTS
>sp|Q6XQH7.2|L_OLVVA RecName: Full=RNA-directed RNA polymerase L; Short=Protein
L;
AltName: Full=Large structural protein; AltName: Full=Replicase;
AltName: Full=Transcriptase
Length=2234

 Score = 30.8 bits (68),  Expect = 0.56, Method: Composition-based stats.
 Identities = 15/34 (44%), Positives = 20/34 (59%), Gaps = 0/34 (0%)
 Frame = -1

Query  126  AERIERPWQVMSELICSGHSAVQVSSHELDGFDP  25
            AE  ER  ++ SE +C     V+VS HE+  FDP
Sbjct  951  AELQERLSELWSEFMCMKIITVEVSLHEIKDFDP  984
```

```
Query= 81

Length=136


                                                                      Score     E
Sequences producing significant alignments:                          (Bits)  Value

sp|Q29293.1|RL3_PIG  RecName: Full=60S ribosomal protein L3        26.6    8.4

ALIGNMENTS
>sp|Q29293.1|RL3_PIG RecName: Full=60S ribosomal protein L3
Length=130

 Score = 26.6 bits (57),  Expect = 8.4, Method: Compositional matrix adjust.
 Identities = 10/19 (53%), Positives = 13/19 (68%), Gaps = 0/19 (0%)
 Frame = +2

Query  47   LEDSAVNRMGRFESYGEVV  103
            L D ++N +GRF  YGEV
Sbjct  106  LSDKSINPLGRFVHYGEVT  124


Query= 82

Length=138


                                                                      Score     E
Sequences producing significant alignments:                          (Bits)  Value


sp|B3PIT8.1|MNMG_CELJU  RecName: Full=tRNA uridine 5-carboxyme...  27.3    6.7

ALIGNMENTS

>sp|B3PIT8.1|MNMG_CELJU
 RecName: Full=tRNA uridine 5-carboxymethylaminomethyl modification
enzyme MnmG; AltName: Full=Glucose-inhibited division
protein A
Length=638

 Score = 27.3 bits (59),  Expect = 6.7, Method: Composition-based stats.
 Identities = 12/20 (60%), Positives = 15/20 (75%), Gaps = 0/20 (0%)
 Frame = +1

Query  70   SLKGEPSSGEYACPQVRVES  129
 SLKGEP +GE A  QV +E+
Sbjct  525  SLKGEPLAGEAAAEQVEIEA  544


Query= 83

Length=130


 Score     E
Sequences producing significant alignments:                          (Bits)  Value


sp|Q1AW66.1|PYRH_RUBXD  RecName: Full=Uridylate kinase; Short=...  27.3    4.7
```

```
ALIGNMENTS
>sp|Q1AW66.1|PYRH_RUBXD RecName: Full=Uridylate kinase; Short=UK; AltName: 
Full=Uridine 
monophosphate kinase; Short=UMP kinase; Short=UMPK
Length=255

 Score = 27.3 bits (59),  Expect = 4.7, Method: Composition-based stats.
 Identities = 16/37 (43%), Positives = 20/37 (54%), Gaps = 3/37 (8%)
 Frame = +3

Query  27   GVTARVQENT---PVRRSGLSPSGPPLRNSG*YGFDP  128
            G +AR  E +   P+RR  L  SG  L  +G YG DP
Sbjct  2    GESARAAETSELGPLRRVVLKLSGESLAGNGGYGIDP  38




Query= 84

Length=116


                                                                   Score     E
Sequences producing significant alignments:                       (Bits)  Value

sp|A4FPQ7.1|RL10_SACEN  RecName: Full=50S ribosomal protein L10    27.7    3.2

ALIGNMENTS
>sp|A4FPQ7.1|RL10_SACEN RecName: Full=50S ribosomal protein L10
Length=177

 Score = 27.7 bits (60),  Expect = 3.2, Method: Composition-based stats.
 Identities = 16/34 (47%), Positives = 21/34 (62%), Gaps = 1/34 (3%)
 Frame = +3

Query  6    MRRHLHCGVTARVQENTPVRRSGLSPSGPAGLNE  107
            +RR L  G T RV +NT V+R+    SG  GL+E
Sbjct  39   LRRALGEGTTYRVAKNTLVKRAA-EQSGVEGLDE  71


Query= 85

Length=103


                                                                   Score     E
Sequences producing significant alignments:                       (Bits)  Value

sp|Q1R0H5.1|RL3_CHRSD  RecName: Full=50S ribosomal protein L3      26.6    5.9
sp|B4SKW3.1|RL3_STRM5  RecName: Full=50S ribosomal protein L3 ...  26.6    6.9

ALIGNMENTS
>sp|Q1R0H5.1|RL3_CHRSD RecName: Full=50S ribosomal protein L3
Length=212

 Score = 26.6 bits (57),  Expect = 5.9, Method: Compositional matrix adjust.
 Identities = 10/22 (45%), Positives = 18/22 (82%), Gaps = 0/22 (0%)
 Frame = -3

Query  74  PERLSGIRSVQPSGHSAVQVSS  9
           P R++ +++V+  G+SAVQV+S
Sbjct  32  PNRVTRVKTVESDGYSAVQVTS  53
```

```
>sp|B4SKW3.1|RL3_STRM5 RecName: Full=50S ribosomal protein L3
 sp|B2FQ45.1|RL3_STRMK RecName: Full=50S ribosomal protein L3
Length=216

 Score = 26.6 bits (57),  Expect = 6.9, Method: Compositional matrix adjust.
 Identities = 10/22 (45%), Positives = 17/22 (77%), Gaps = 0/22 (0%)
 Frame = -3

Query  77  VPERLSGIRSVQPSGHSAVQVS  12
             P R++ I++V+  G+SAVQV+
Sbjct  35  TPNRIAQIKTVETDGYSAVQVT  56


Query= 86

Length=104


                                                                      Score     E
Sequences producing significant alignments:                          (Bits)  Value

sp|Q5MI53.1|TRPA_LEPBP  RecName: Full=Tryptophan synthase alph...  26.9    4.9

ALIGNMENTS
>sp|Q5MI53.1|TRPA_LEPBP RecName: Full=Tryptophan synthase alpha chain
 sp|B0SDM7.1|TRPA_LEPBA RecName: Full=Tryptophan synthase alpha chain
Length=266

 Score = 26.9 bits (58),  Expect = 4.9, Method: Composition-based stats.
 Identities = 11/23 (48%), Positives = 13/23 (57%), Gaps = 0/23 (0%)
 Frame = +3

Query  21   HCGVTARLD*TNPRQVVGWAVPD  89
            HCG+T  LD      VVG  +PD
Sbjct  106  HCGITKFLDNAKDSGVVGLVIPD  128


Lambda      K        H
   0.318    0.134    0.401
Gapped
Lambda      K        H
   0.267   0.0410    0.140
Matrix: BLOSUM62
Gap Penalties: Existence: 11, Extension: 1
Number of Sequences: 454086
Number of Hits to DB: 13772125358
Number of extensions: 328258447
Number of successful extensions: 452965
Number of sequences better than 10: 744
Number of HSP's better than 10 without gapping: 0
Number of HSP's gapped: 452955
Number of HSP's successfully gapped: 1292
Length of database: 169309753
T: 12
A: 40
X1: 16 (7.3 bits)
X2: 38 (14.6 bits)
X3: 64 (24.7 bits)
S1: 41 (20.4 bits)
ka-blk-alpha gapped: 1.9
ka-blk-alpha ungapped: 0.7916
ka-blk-alpha_v gapped: 42.6028
ka-blk-alpha_v ungapped: 4.96466
```

```
ka-blk-sigma gapped: 43.6362
```
