# Supplementary material for: tRNA Core Hypothesis for the Transition from the RNA World to the Ribonucleoprotein World
Source: Life (Basel). 2016 Mar 23;6(2):15. doi: 10.3390/life6020015 (PMC4931452; doi:10.3390/life6020015)
Supplement: Supplementary file 1 [file life-06-00015-s001.zip › Supplementary information 1.pdf]

Supplementary Materials: tRNA Core Hypothesis for the Transition from the RNA World to the Ribonucleoprotein World

Savio T. de Farias [1,†,*], Thais G. Rêgo [2,†] and Marco V. José [3,†,*]

Ancestral sequence of cognate tRNAs for:

ile = "GCCGCTCAGGAACTCGGGCTAATACGGGTTCGACCCCCT"
asn =“CTGTAGCTGGAAGATTCGGCTGTTAACCGAATGGGTAGGTTCGAATCCTACGGCGA"
val = "GTTTAGGATCACGTCCCTAACAGCAGAAGGTCCTTGAAGGGTGAACC"
asp = "GAGTTCAGGAGAATACGCCTGTCCGCAGGTCCGGGTTGAGTCCCTCCGGACC"
gly = "CAGATAAGTTCAGACATAACTTGCCAAGGTCGTTCGATTCGTTCTGC"
ser = "GGCTGGACTGAACGAATCCCCGACA"
thr = "AGTGGTGGGCTGGGCCGTACCAGATGGATCGAAGCCATCT"
ala = "AGCTCATGAGACGACACTTGCACTGCGGAGTGACCGCTA"
